# Phosphorothioated amino-AS1411 aptamer functionalized stealth nanoliposome accelerates bio-therapeutic threshold of apigenin in neoplastic rat liver: a mechanistic approach

**DOI:** 10.1186/s12951-022-01764-4

**Published:** 2023-01-25

**Authors:** Moumita Dhara, Ashique Al Hoque, Ramkrishna Sen, Debasmita Dutta, Biswajit Mukherjee, Brahamacharry Paul, Soumik Laha

**Affiliations:** 1 Department of Pharmaceutical Technology, Jadavpur University, Kolkata, 700032 India; 2 Department of Coatings and Polymeric Materials, North Dakota State University, Fargo, USA; 3 Dana Farber Cancer Institute, Boston, MA USA; 4 Harvard Medical School, Boston, MA USA; 5 Central Instrument Facility, CSIR-Indian Institute of Chemical Biology, Kolkata, 700032 India

**Keywords:** Aptamer, Apigenin, Stealth nanoliposomes, Intratumor drug accumulation, Apoptosis, Pharmacokinetics

## Abstract

Hepatocellular carcinoma (HCC) is a leading cause of death globally. Even though the progressive invention of some very potent therapeutics has been seen, the success is limited due to the chemotherapeutic resistance and recurrence in HCC. Advanced targeted treatment options like immunotherapy, molecular therapy or surface-engineered nanotherapeutics could offer the benefits here owing to drug resistance over tumor heterogenicity. We have developed tumor-sensing phosphorothioate and amino-modified aptamer (AS1411)-conjugated stealth nanoliposomes, encapsulating with apigenin for precise and significant biodistribution of apigenin into the target tumor to exploit maximum bio-therapeutic assistances. The stable aptamer functionalized PEGylated nanoliposomes (Apt-NLCs) had an average vesicle size of 100–150 nm, a smooth surface, and an intact lamellarity, as ensured by DLS, FESEM, AFM, and Cryo-TEM. This study has specified in vitro process of optimum drug (apigenin) extrusion into the cancer cells by nucleolin receptor-mediated cellular internalization when delivered through modified AS1411 functionalized PEGylated nanoliposomes and ensured irreversible DNA damage in HCC. Significant improvement in cancer cell apoptosis in animal models, due to reduced clearance and higher intratumor drug accumulation along with almost nominal toxic effect in liver, strongly supports the therapeutic potential of aptamer-conjugated PEGylated nanoliposomes compared to the nonconjugated formulations in HCC. The study has established a robust superiority of modified AS1411 functionalized PEGylated nanoliposomes as an alternative drug delivery approach with momentous reduction of HCC tumor incidences.

**Graphical Abstract:**

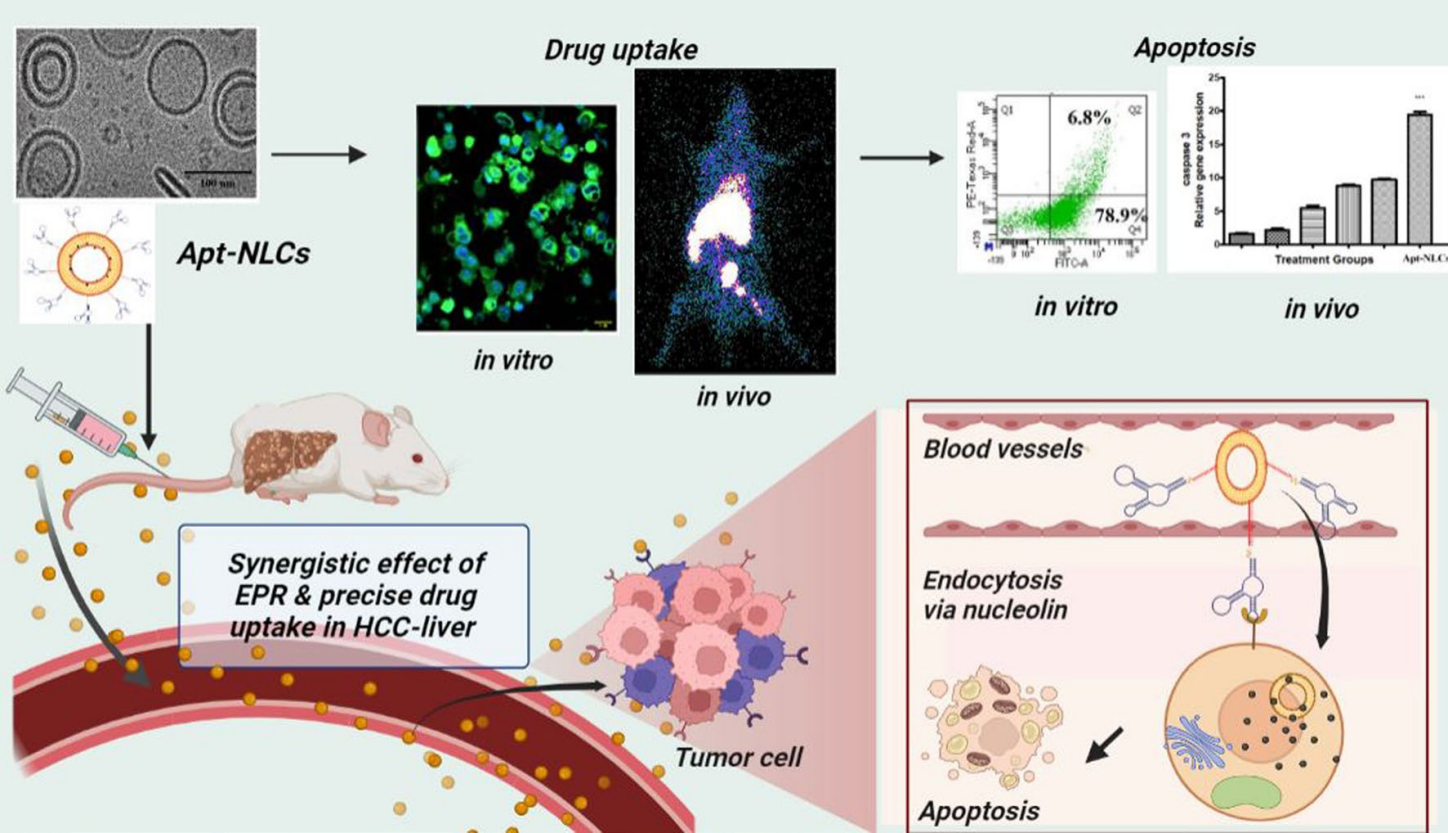

**Supplementary Information:**

The online version contains supplementary material available at 10.1186/s12951-022-01764-4.

## Introduction

Despite some landmark development in chemotherapy, molecular or immunotherapy, treating or curing hepatocellular carcinoma (HCC) is still a global health issue, and the shocking fact is future estimated incidences of hepatic malignancy that could cross one million by 2025 [1]. The development of more and more potential, alternative, and cost-effective therapeutics is on-demand to combat this dynamic challenge. Naturally occurring flavonoids, such as apigenin, quercetin, and many having enormous potential as anticancer agents, have already been tried to formulate by encapsulating in nanoparticles owing to their poor solubility and bioavailability [2]. But in clinics, therapeutic challenges such as lack of specificity toward the target organ and desired curing response along with bare minimum side effects to the normal or healthy cells are yet to be adequately addressed [3]. Optimizing precise therapeutic accumulation to the target site with ligand-functionalized nanoformulations could minimize drug resistance and improve therapeutic tolerances [4]. Thus, it can accelerate their scope for preclinical to clinical translation [5]. Apigenin has been well demonstrated both in vitro and in vivo, promoting apoptosis, inducing cell cycle arrest, suppressing the cancer cell invasion along with its autophagy and immunogenic activities [6]. Apigenin-encapsulated nanoparticles were already applied effectively for delaying the progression of different types of cancer, including HCC [7]. However, along with refining solubility and bio-availability, precise delivery through ligand-functionalized lipid nanocarriers could improve the apoptotic potential of apigenin in a great extent [8]. Aptamer fabricated nano liposomal drug delivery is already in prime focus relating to advanced targeted drug delivery for cancer [9]. Further, apigenin-loaded nano liposomes modified with ligand-targeted drug delivery system have not been explored yet, for HCC. Formulation optimizations, the process of incremental biotherapeutic accumulation in tumor cells, and related apoptosis still need scientific clarity. We hypothesize that the therapeutic threshold of apigenin might be uniquely upgraded by precise, predictable, and effective drug accumulation to the target, cancerous hepatocytes, applying stable, aptamer functionalized PEGylated nanoliposomes.

AS1411 is a non-immunogenic, thermostable 26-mer guanine-rich DNA aptamer with quadruplex structural advantages, which provides exceptional affinity toward the nucleolin proteins overexpressed on the surface of HCC cells [10]. Customized phosphorothioate backbone modification in amino-modified DNA aptamer (AS1411) offers superior pharmacological stability to the aptamer and is used for the effective target-specific nano-drug delivery system [11]. DSPE-PEG is intended to provide a longer half-life in vivo with reduced clearance due to its stealthy coating over the nano liposomal surface [12].

Here, we have developed apigenin-encapsulated, PEGylated nanoliposomes functionalized with phosphorothioated amino-modified-AS1411 aptamer. We explored the process of incremental drug internalization vs apoptosis through dual functionalized aptamer conjugated PEG-nanoliposomes over pegylated (non-aptamer conjugated) or normal nanoliposomes for apigenin both in vivo and in vitro models. Finally, we concluded the therapeutic benefits and tried to establish the additional biological evidence mostly relevant to the preclinical or clinical research and outcome.

## Experimental section

### Materials

Apigenin was obtained from Sigma–Aldrich Lab. (MO, USA). Carboxymethyl-PEG2000-1,2-Distearoyl-sn-glycero-3-phosphoethanolamine or (DSPE-PEG2000-COOH) was purchased from Laysan Bio Inc. (AL, USA). 1-Ethyl-3-(3-dimethylamino propyl) carbodiimide, chloride (EDC, HCl), *N*-hydroxy succinimide (NHS), cholesterol, dimethyl sulfoxide, butylated hydroxytoluene (BHT), ethanol, chloroform, sodium chloride, potassium dihydrogen phosphate, and disodium hydrogen phosphate were procured from E Merck Specialties Ltd. (Mumbai, India). Fluorescein isothiocyanate (FITC) and soya-l-α-lecithin (SPC) were acquired from Hi-Media Lab. Pvt. Ltd. (Mumbai, India).

The 26-mer- AS1411 DNA aptamer, having sequence 5′-GT GGT GGT GGT TGT GGT GGT GGT GG-3′ (molecular weight: 8674.3 g/mole) was custom-synthesized as phosphorothioate backbone and 3′-amino-modified form by GCC Biotech (Kolkata, India).

### Cell lines

All the in vitro cell-based studies were conducted on human liver cancer cell lines, Hep-G2/Huh-7 cell lines purchased from NCCS (National Centre for Cell Science, Pune, India). The cell lines were sub-cultured in DMEM media, supplemented with 10% fetal bovine serum (FBS) and 1% antibiotic solution, and maintained at 5% CO_2_ containing humidified air at 37 °C.

Hep G2 is a well-differentiated immortal hepatocellular carcinoma cell line obtained from a Caucasian male aged 15 years [13]. The popularity of the use of Hep G2 cells for in vitro cellular study is related to the fact that it is primary liver cancer (well represented by hepatocellular carcinoma, HCC). Since Hep G2 cells have a high degree of morphological and functional differentiation in vitro, they are considered a suitable model for studying the intracellular trafficking and dynamics of various chemicals, including drugs, formulations, proteins, and ligand molecules in vitro [14]. Several studies reported Hep G2 as in vitro model for detecting cytoprotective, cytotoxic, genotoxic, and antigenotoxic agents in hepatocarcinogenesis and drug-targeting investigations [15, 16]. Thus, we selected Hep G2 cells for the in vitro investigation.

Huh 7 is also a well-differentiated hepatocellular carcinoma cell line, isolated from the tumor originated from liver hepatocytes, from a 57-year-old Japanese male.

### Sprague Dawley (SD) rats for hepatocellular carcinoma (HCC) model

Sprague Dawley (SD) rats (120–150 g) were procured from NIN (National Institution of Nutrition), Hyderabad, India, and animal studies, conducted here were approved by the Institutional Animal Ethical Committee (AEC), Jadavpur University on submission of proper experimental protocol plan following guidelines of the Purpose of Control and Supervision of Experiments on Animals (CPCSEA), Government of India.

### Pre-formulation studies (FTIR-Fourier-transform infrared spectrometry)

Fourier -transform infrared spectrometric (FTIR) analysis was applied to examine if there was any molecular interaction between the drug and the excipients used during the formulation development [17]. FTIR spectroscopy was carried out with the drug (pure apigenin, Api), drug excipients (SLY, CHC, DSPE-PEG, BHT) individually along with their physical mixture, formulated plain nanoliposome (NLCs) with or without the drug, PEG-NLCs and finally, aptamer conjugated PEGylated nanoliposomes, Apt-NLCs, within the wave number array of 4000–400 cm^−1^ under inert atmospheric conditioning KBr palate by an FTIR spectrophotometer (Bruker-(Alpha), Ettlingen, Germany) and were analyzed.

### Preparation of apigenin (Api)-loaded nanoliposomes (NLCs, PEG-NLCs) and aptamer functionalized PEGylated Api-loaded nanoliposomes (Apt-NLCs)

Apigenin-encapsulated plain nanoliposomes (NLCs) and PEGylated NLCs were prepared by the thin-film hydration method [18]. For subsequent preparation of the aptamer functionalized PEGylated nanoliposomes (Apt-NLCs), we have used the PEGylated NLCs (PEG-NLCs). The coupling of aptamer on the liposomal surface was accomplished by a covalent attachment of the amino group of the aptamer (NH_2_-modified AS1411) with the carboxyl group of PEG-DSPE-COOH in the PEG-NLCS [19]. Surface grafting of amino-modified AS1411 aptamer on the prepared pegylated NLCs was conducted through the activation carboxyl group of PEG component (PEGylated-1,2-Distearoyl-sn-glycero-3-phosphoethanolamine (PEG-DSPE-COOH)). The pegylated NLCs were suspended in a mixture of 150 mM *N*-hydroxy succinimide (NHS) and 300 mM ethyl (dimethyl aminopropyl) carbodiimide (EDC) at room temperature for 1 h by maintaining all through a neutral of 1 M Tris HCI buffer [19, 20]. The resulting solution was added to 25 µl aliquot from 100 µM of 3′amine-modified AS1411 aptamer stock solution (the final concentration of AS1411 in experimental nanoliposomes Apt-NLCs was 5 µM) [21]. Here, the portion of the sample aptamer solution was previously arranged by applying a denaturation–renaturation process so that it would interact in a biochemical reaction readily. The solution mixture was kept in a shaker incubator overnight at normal room temperature to accomplish aptamer ligation on the surface of PEGylated NLCs. Finally, we removed the excess and unconjugated aptamer from aptamer-conjugated nanoliposomes (Apt-NLCs) by centrifuging and washing the mixture thrice with nuclease-free deionized water and the aptamer conjugated formulations were stored at 4 °C for further analysis [22].

The blank aptamer conjugated liposomes (blank-Apt-NLCs) were also prepared following a similar process but without encapsulating apigenin in the liposomes. Both NLCs and Apt-NLCs were labeled with FITC (fluorescein isothiocyanate) dye by incorporating 10 µl of FITC (0.4% w/v ethanolic solution) into the organic phase during thin layer formation of liposome preparation [22].

### Determination of aptamer conjugation by agarose gel electrophoresis

To determine the successful ligation of aptamer AS1411 with the PEGylated liposomes (PEG-NLCs), aptamer conjugated nanoliposome (Apt-NLCs), free aptamer (AS1411), and a commercial 50 bp DNA ladder were sequentially placed in the wells of previously prepared 1% agarose gel plate. Through running electrophoresis for 20 min at 100 V, we observed the migration pattern for different samples as mentioned above [22, 23]. Here, 0.5 mg/ml ethidium bromide was used in the agarose gel to visualize bands in the gel electrophoresis, and gel loading dye was used for staining DNA during the electrophoresis process. Finally, free DNA aptamer, DNA ladder, and the DNA-conjugated nanoformulations in gel plate were visualized using a UV transilluminator as reported earlier [24].

To determine the conjugation of AS1411 aptamer on to PEG-NLCs quantitatively, first we repetitively washed out (more than three times) the Apt-PEG-NLCs solution with nuclease free water to remove all the unconjugated free aptamer available in Apt-PEG-NLCs. Finally, with proper dilution adding nuclease free water to it, we compared the amount of AS1411 aptamer in aptamer conjugated nanoliposomes, Apt-PEG-NLCs along with 1 µl of a free AS1411 (DNA aptamer) and a plain PEGylated nanoliposomes, PEG-NLCs (without aptamer) by applying nanodrop UV spectrophotometric (nano 300 micro spectrophotometer, IGene Labserve Pvt Ltd. India) method [25, 26].

### Percentage of drug loading and drug entrapment efficiency

To determine drug-loading, the accurately weight of lyophilized liposome (2 mg), NLCs/PEG-NLCs or Apt-NLCs was dissolved in 2 ml of ethanol-acetonitrile-dimethyl sulfoxide solvent mixture at a ratio of 0.5:1:1(v/v) as the best suit solvent composition for testing drug loading of the experimental formulations. It was determined by the trial-and-error method. The resulting solution was vortex-mixed for 1–2 h and then centrifuged for 20 min at 10,000 rpm, clear supernatant (1 ml) was collected to measure the absorbance intensity by the UV/VIS-spectrometer at the corresponding λ_max_ of apigenin 340 nm, and the drug content in the test liposomes were analyzed from the corresponding standard calibration curve prepared early [7, 18]. Thus, percentage drug-loading and drug encapsulation efficacy were obtained using the formulae mentioned below.

1 $${\text{\% Drug loading = }}\left( {\text{Amount of drug in liposome/Amount of liposome used}} \right) \times { 100}$$

2 $${\text{\%  Yield = }}\left( {\text{Weight of dry powdered liposome/Total weight of all the components used in the formulation}} \right) \, \times { 100}$$

### Particle size distribution & ζ-potential measurements

Particle size distribution and ζ-potential of NLCs, PEG-NLCs, and Apt-NLCs were analyzed by Malvern Zetasizer Nano-ZS 90; (Malvern Instruments, UK) applying dynamic light scattering (DLS) technique with sample suspension of the experimental liposomes prepared by diluting them properly, vortex-mixing and sonication process. The results represented were the average particle size considering the standard deviation of at least three different batches of the experimental liposomes.

### Field emission scanning electron microscopy (FESEM)

Surface morphology of prepared test nanoliposomes (NLCs, PEG-NLCs, and Apt-NLCs) were observed under FESEM by forming a very thin layer of samples coated with platinum with a platinum coater at an accelerating voltage of 10 kV [27].

### Atomic force microscopy (AFM)

The three-dimensional architecture of Apt-NLCs was observed under atomic force microscopy (AFM) by placing one drop of properly diluted and air-dried formulation suspension on a mica surface coverslip plate. Thus, shapes of the Apt-NLCs were observed through AFM by fixing a resonance frequency at 150–250 kHz under ACAFM mode [28, 29].

### Cryo-transmission electron microscopy (Cryo-TEM)

The internal bilayer morphology, membrane stability and surface functionalization characteristics of the prepared experimental nanoliposome, Apt-NLCs, were determined by cryo-TEM. Here test liposomes were dispersed (100 µg/ml) in Milli Q water, and an aliquot (4 μl) of it was placed on a glow-discharged 300 mesh carbon-coated copper grid (TEM grid), and the air-dried samples were visualized under the cryo-TEM instrument (200 kV Talos-Arctica electron microscope, FEI/Thermo Scientific) [30].

### In vitro drug release

In vitro drug release study was conducted by taking 2 ml of experimental liposomal suspensions (which were prepared by dispersing 2 mg of lyophilized NLCs, PEG-NLCs, and Apt-NLCs individually in 2 ml of buffer solution, PBS phosphate buffer with pH 7.4/acetate buffer, with pH-5) into a dialysis bag (Dialysis Membrane-110, Himedia, India). Then, the sample was dialyzed into 50 ml of respective drug release media, PBS/acetic buffer, containing 0.01% (w/v) β-cyclodextrin at room temperature (37 °C) by placing the dialysis system on a magnetic stirrer with very slow stirring at 100 rpm. During each study, 1 ml of drug release media was collected from 50 ml of solution at predetermined intervals up to 96 h, and was replaced by 1 ml of fresh media immediately [31]. Finally, all the samples were analyzed by UV–VIS spectroscopy at 340 nm wavelength against the respective medium without drug, and drug concentration was determined using a calibration curve prepared earlier. In vitro cumulative drug release data were represented through various release kinetic models such as zero order, first order, Higuchi model, Korsmeyer–Peppas and Hixson–Crowell models, and the highest *R*^2^ (correlation coefficient) value was evaluated after plotting the drug released data to determine the best suit drug-release kinetic model [32].

### Stability study

Stability assay of the intended experimental formulation, aptamer-conjugated PEGylated nanoliposomes (Apt-NLCs), was performed as per ICH guidelines [22], at the storage conditions (40 ± 2 °C and 75 ± 5% RH) and in the refrigerated form at (~ 4 °C) for 6 months. The samples were then subjected to examination for any changes in particle size or morphology using FESEM and variation in drug content in the prepared nanoliposome (Apt-NLCs) by conducting a drug loading assay.

### MTT assay to evaluate in vitro antiproliferative activity of test liposomes

Two different liver cancer cell lines, Hep G2 and Huh-7, were procured from the National Centre for Cell Science, Pune, India. The cells were cultured in DMEM media, maintaining all necessary conditions [33]. The cell (taking 1 × 10^3^ cells/well) viability expressed as IC_50_ doses for the free drug (apigenin), NLCs/PEG-NLCS, Apt -NLCs and blank-Apt-NLCs (Apt -NLCs without the drug) in both the cell lines were evaluated through MTT [(4,5-dimethylthiazol-2-yl)-2,5-dimethyltetrazolium bromide] assay using a wide range of individual equivalent drug concentration (1 µM, 10 µM, 20 µM, 30 µM, 40 µM, 50 µM) following the standard protocol [34].

### Human peripheral blood mononuclear cell (PBMC) processing

This process is performed by isolating peripheral blood mononuclear cells (PBMC) from anticoagulated blood. Anticoagulated blood was added with an equal volume of Ficoll-Hypaque (Histopaque-1077) and centrifuged at 400*g* for 30 min to separate the cells from the whole blood. The PBMCs were collected from the interface of two liquids, washed with PBS (0.01 M, pH 7.4) twice, and resuspended in the RPMI 1640 medium [22]. The cytotoxic activity of different nanoliposomes suspended in PBS in the presence of a very minute amount of dimethyl sulfoxide, DMSO (final DMSO concentration < 0.1%), was evaluated for PBMC using an MTT assay.

### Apoptosis assay

To verify the apoptotic potential of aptamer functionalized nanoliposome, Apt-NLCs, in comparison with the nonconjugated ones (NLCs/Peg-NLCs) in Hep G2 cells, we executed a flow cytometric (BD LSRFortessa™, BD Biosciences) study using FITC-Annexin V staining assay kit (BD Biosciences). The apoptotic activity of the experimental nanoliposomes containing an equivalent amount of active drug (considering the IC_50_ values from MTT) was determined upon 48 h of prior treatment and following the protocols [29].

### Determination of cell-targeting potential of the aptamer (AS1411) assessing in vitro cellular uptake of the experimental nanoliposomes

In vitro uptake of FITC-labeled PEG-NLCs and FITC-labeled Apt-NLCs by HepG2 cells treated with the formulations containing the equivalent amount of drug was evaluated quantitatively at the three successive time points (1 h, 2 h, 4 h) by flow cytometric method. To confirm the receptor-mediated cellular uptake of an aptamer (AS1411) functionalized PEGylated nano-liposome (Apt-NLCs) over the other non-functionalized pegylated nanoliposome (PEG-NLCs), a competitive assay was performed. In this competitive assay, Hep G2 cells were preincubated with an excess amount (0.2 µg/well) of free aptamer (AS1411) for 2 h to block available coupling receptors (majorly nucleolin) [34, 35]. After that, it was treated with Apt-NLCs maintaining all other conditions the same. A quantitative comparison of in vitro cellular uptake between FITC-labeled formulations PEG-NLCs and Apt-NLCs in Hep G2 cells and FITC-labeled Apt-NLCs against aptamer-pretreated Hep G2 cells were estimated to verify receptor-mediated cellular uptake of aptamer-conjugated experimental nanoliposomes, Apt-NLCs. FACS Diva software (BD Biosciences) was used to analyze the data [35]. We have also captured confocal microscopic (Olympus Fluo View FV10i, Olympus) images of Hep G2 cells treated with FITC-labeled PEGylated-NLCs and -Apt-NLCs at 1 h and 4 h intervals to visualize the cellular uptake qualitatively.

### Study of cell cycle arrest and apoptosis-related proteins

FACS (BD Biosciences, USA) analyzer was also used to analyze different phases of cell cycle during cell propagation, referring to apoptotic behavior of test nanoliposomes (an equivalent IC_50_ dose of apigenin containing in the respective amount of NLCs, PEG-NLCs and Apt-NLCs was used to treat 1.5 × 10^5^ Hep G2 cells per well in six-well plates for 24 h). After washing in 50 µl RNase-free water and treating with Anexin-V, propidium iodide (PI) solution, the cells were subjected to analysis [36].

Similarly, apoptosis-related signaling proteins (p53, cleaved caspases, Bcl-2) were also estimated using respective protein markers by flow cytometric assay. These proteins have a major influence on the genetic regulation process (DNA synthesis, damage, or repair) during apoptosis. The experiments were conducted in Hep G2 cells, treated with Apt-NLCs, PEG-NLCs, and NLCs, taking an equivalent dose of apigenin for 24 h following the published protocol and guidelines [36].

### Development of DENA-induced hepatocellular carcinoma in Sprague Dawley (SD) rats

The Institutional Animal Ethical Committee Jadavpur University approved all the animal studies. Development of hepatocellular carcinoma induced by chemical carcinogens such as diethyl nitrosamine and 2-acetylaminofluorine (AAF) has been well-known in animal models for the last several decades [37, 38]. In the present study, we used Sprague Dawley rats. Carcinogen-induced initiation-promotion model was used. Hepatocellular carcinoma (HCC) was developed in Sprague Dawley male rats using chemical carcinogens, applying initially a single intraperitoneal dose of 200 mg/kg of diethyl-nitrosamine (DENA), followed by a bi-weekly oral administration of 0.5% of w/w 2-acetylaminofluorene (2-AAF) for 16 weeks [38, 39]. Experimental groupings (A–G) were done as normal control rats (Group A), carcinogen control rats (Group B), carcinogen-treated rats received free apigenin (Group C), carcinogen-treated received NLCs (Group D), carcinogen-treated rats received PEG-NLCs (Group E), carcinogen-treated rats received Apt-NLCs (Group F), and normal rats received Apt-NLCs (Group G). All the carcinogen-treated groups were injected (through the i.v. route) 20 mg/kg body weight of free apigenin or an equivalent dose of the experimental formulations once a week for 8 weeks after the 16th week of carcinogen-treated animals [7, 22].

### Pharmacokinetic study

The plasma and liver pharmacokinetic profiles for free drug, NLCs, PEG-NLCs, and Apt-NLCs were evaluated in HCC-induced Sprague Dawley (SD) rats (body weight, 125–150 g). Plasma and liver samples were collected from the experimental animals after injecting a single i.v. bolus dosage of 2 mg/kg of body weight of apigenin or corresponding amount of NLCs, PEG-NLCs, and Apt-NLCs containing equivalent apigenin, at 2, 4, 8, 12, 24, 48, 72, 96, and 120 h. The final analytical samples were prepared from the biological samples through the liquid–liquid extraction process (using TBME, t-butyl methyl ether as a volatile solvent for drug extraction from plasma and liver homogenate) and analyzed by LC–MS/MS method applying naringenin as an internal standard [22]. The experiments were repeated in triplicate.

### Assessing biodistribution of NLCs, PEG-NLCs, and Apt-NLCs by Gamma scintigraphy study

To investigate the accumulation efficiency of liposomes, NLCs, PEG-NLCs and Apt-NLCs, toward the target organ (liver), the formulations were radiolabeled by technetium-99 m (^99m^Tc) following the stannous chloride reduction method with a radiolabeling accuracy almost above 90% [33]. Radiolabeled formulations (equivalent drug dose) were injected through the cannulation process to carcinogen-treated rats to ascertain the pattern of bio-distribution of different test nanoliposomes (NLCs/PEG-NLCs/Apt-NLCs) at 4 h and 8 h after their administrations. The results were articulated as % administered dose distributed per gram (%ID/g) of tissues or organs. Gama-scintigraph images of the animals were captured at 4 h and 8 h post-injection through GE Infinia γ Camera facilitated along with Xeleris Work Station, USA [29, 33].

### In vivo hepatocyte uptake of fluorescent-labeled formulations by confocal microscopic study

To elucidate the accumulation pattern of different test nanoliposomes in the neoplastic hepatocytes, we have injected FITC-labeled NLCs, PEG-NLCs, and Apt-NLCs into the carcinogen-induced HCC animals through the tail veins. Taking a time interval of 4 h and 8 h post-injection, tumors and tumor-adjacent tissues were collected by sacrificing the animals and stored at 10% formaldehyde [22]. The tumors and tumor-adjacent tissue sections were fixed on slides and evaluated under confocal microscopy.

### Assessing antitumor efficacy of the experimental nanoliposomes using a prepared animal model

To ascertain the antitumor potencial of the test nanoliposomes, we divided the animals into seven groups, Gr.A, normal (control), Gr. B, HCC positive control animals, HCC control, Gr.C, HCC induced animals treated with the free drug, Gr.D, HCC induced animals treated with NLCs, Gr.E, HCC induced animals treated with PEG-NLCs, Gr. F, HCC-induced animals treated with Apt-NLCs, Gr G, normal animals treated with Apt-NLCs. In the case of Gr C, D, E, F, and G, we followed a treatment schedule of once-weekly i.v. dose of 20 mg/kg body weight of free apigenin or equivalent amount formulations for twelve weeks. There were seven experimental animals in each group. At the end of the treatment, we collected the whole liver, and liver sections from the sacrificed experimental animals were assessed for the degree of antitumor potency of Apt-NLCs compared to NLCs/PEG-NLCs or free drug.

### Gross and histopathological examination for liver morphology

We performed a gross macroscopic examination of the whole liver to identify the tumor incidences. Further, microscopic observation of tumor tissue sections by histopathological staining (with hematoxylin and eosin, periodic acid-Schiff) was conducted to evaluate the microscopic changes in the liver histopathology and to identify hepatic altered focal lesions [7].

### Identifying apoptotic gene expression level through the qRT-PCR analysis

Total RNA was extracted from frozen (stored at − 70 °C) liver tissue sample (from Gr B, C, D, E & F animals) with Trizol reagent and was evaluated through nanodrop (QI Aexpert) after following the manufacturer’s protocol. Then, with the required amount of sample RNA, cDNA was prepared and using this template, RT-PCR studies were performed with the aid Bio-Rad SYBR green PCR master mix along with corresponding primers as per manufacturer’s instructions [40, 41]. The specific synthetic primers (for *p*^53^, F:5′-ATGTTTTGCCAACTGGCCAAG-3′, R:5′-TGAGCAGCGCTCATGGTG-3′); (for caspase 3, F: 5′-GTGGAACTGACGATGATATGGC-3′, R: 5′-CGCAAAGTGACTGGATGAACC-3′); (for Bcl-2, F:5′-TGTGGATGACTGACTACCTGAACC-3′, R:5′-CAGCCAGGAGAAATCAAACAGAGG-3′) and (for β-actin, F:5'-AAGATCCTGACCGAGCGTGG-3′, R:5′-CAGCACTGTGTTGGCATAGAGG-3′) were purchased from Saha Gene, Hyderabad, India and maintaining the thermo-cycling conditions as per the manufacturer’s protocol. The qRT-PCR studies were performed on Rotorgene (Qiagen) instrument. The fluorescence activity in qRT-PCR demonstrated the relative gene expression level for corresponding target genes and it was determined by applying 2-∆∆Ct calculative process, where β-actin was taken as the housekeeping gene [42].

### Hepatic function test

Blood samples were collected from all the groups of experimental animals (Gr A-G), and after processing as per the reported method [33], the level of AST (aspartate aminotransferase), ALT (alanine transaminase), and ALP (alkaline phosphatase) were determined using commercially available bioassay kits (Coral Clinical Systems, Goa, India) following manufacturer protocols [33].

### Statistical analysis

The data were statistically analyzed using ORIGIN 8.0 software, one-way ANOVA followed by post hoc Dunnett’s test using Graph Pad Prism Software, considered 5.0. p < 0.05 was as a minimum level of significance.

## Results and discussion

### Physical characterization

#### Pre-formulation and preparation of NLCs/PEG-NLCs/Apt-NLCs

**Fig. 1:**
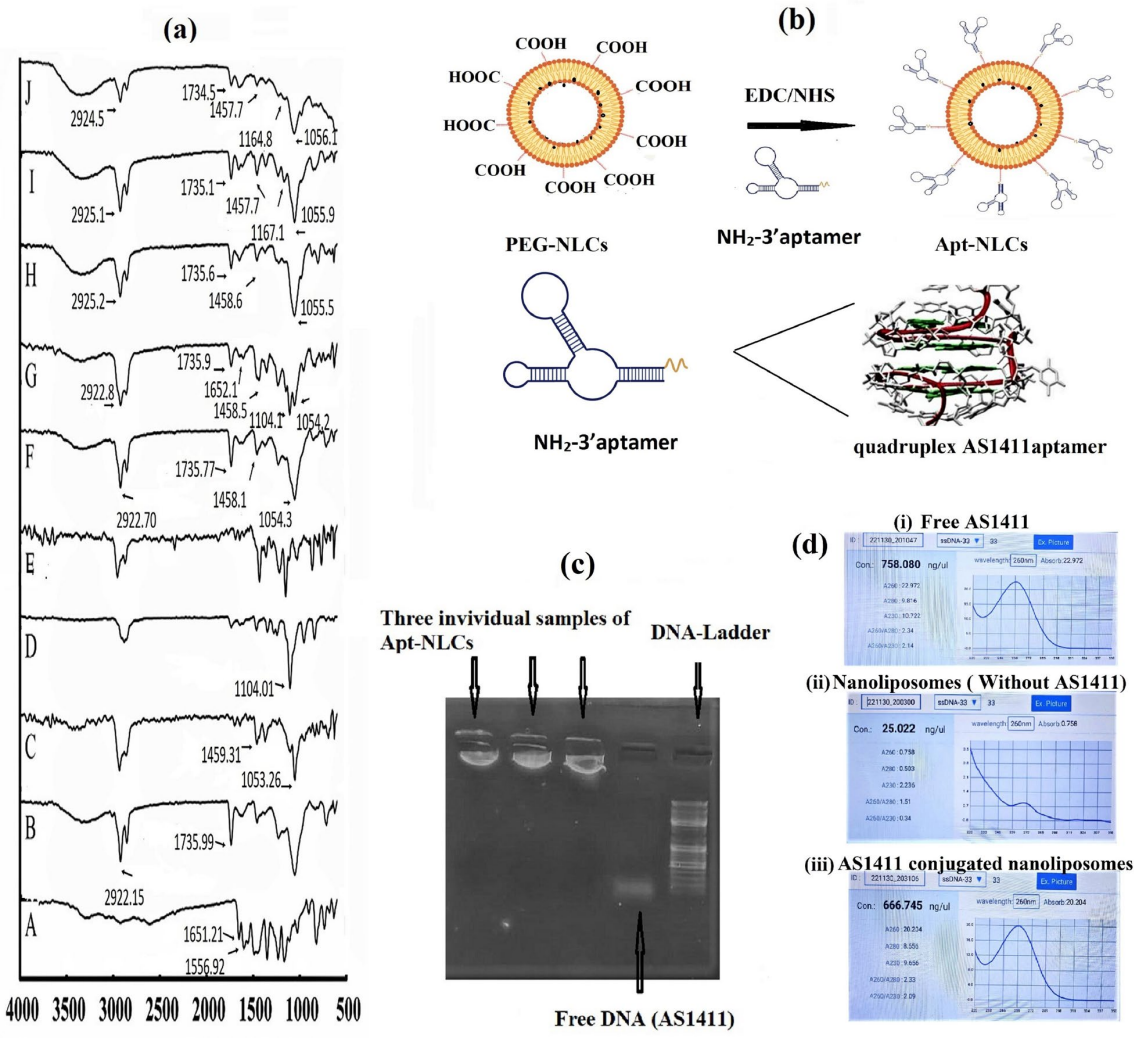
Drug-excipients interaction and aptamer conjugation on stealth nanoliposome. **a** FTIR spectra of apigenin (A), soya lecithin (B), cholesterol (C), DSPE-PEG-2000-COOH (D), BHT (E), Blank formulation (F), the physical mixture of all constituents (G), NLCs (H), PEG-NLCs (I), and Apt-NLCs (J); **b** Pictorial representation of surface functionalization of PEG-NLCs with modified NH2-AS1411; **c** Determination of aptamer conjugation by agarose gel. The first three lanes from the left had (Apt-NLCs) showing no sample run, the 4th lane showed free aptamer (AS1411) showing movement, and the 5th lane had DNA ladder; **d** i, ii, iii represented spectrometric reading for free AS1411, plain nanoliposomes AS1411 conjugated nanoliposomes by nanodrop UV spectrophotometer, (experiment was conducted on triplicate set)

Thin film hydration method was followed for the preparation of experimental nanoliposomes. FTIR spectra for apigenin (Api), each of the excipients (SLY, CHC, DSPE-PEG, BHT), their physical mixture with drug, along with test nanoliposomes NLCs, Apt-NLCs with Apigenin (Api) (Fig. 1a) showed the presence of their characteristic functional groups, for Api (C=C stretching at 1556.92 cm^−1^and C=O stretching at 1651.21 cm^−1^); for SLY (C=O stretching at 1735.99 cm^−1^ and C–H stretching at 2922.15 cm^−1^); for CHC (C–H bending at 1459.31 cm^−1^and C–O stretching at 1053.26 cm^−1^); and for DSPE-PEG (2000)-COOH (C–N medium intensity stretching at 1104.01 cm^−1^) in their physical mixture and in the formulations as in original components, suggesting that nanoliposomes were formulated successfully without any chemical interaction among the ingredients. However, minor shifting of bands (H–O stretching) suggests the formation of weak Van der Waal interaction or weak hydrogen bonds due to physical interaction during formulation development. The characteristic peak of apigenin was absent in the NLCs without the active drug (called here Blank formulation). It was also missing in the prepared test nanoformulations (both in NLCs and Apt-NLCs), indicating complete drug encapsulation. Thus, we confirmed the chemical compatibility of apigenin and other excipients using an FTIR study and successful drug encapsulation in the developed nanoliposomes (NLCs, PEG-NLCs, and Apt-NLCs).

#### Determination of aptamer conjugation

The successful conjugation of phosphorothioated amino-modified AS1411(DNA-aptamer) with PEG-NLCs was determined using agarose gel-electrophoresis by observing prominent fluorescence bands under UV-transilluminator. Figure 1c depicted a gel-electrophoresis assay, and the migration of parallel luminating bands for 50 bp commercial DNA ladder was noticed clearly. Free DNA (AS1411) was also migrated near 25 bp position of 50 bp commercial DNA ladder, while little or no migration for aptamer-conjugated nanoliposomes (Apt-NLCs) was observed. The three stable luminating bands near the starting wells for three individual samples of Apt-NLCs from three different batches indicated successful DNA conjugation with the nanoformulations. Aptamer-conjugated DNA could not migrate through the agarose gel plate with the same speed of free aptamer as it was covalently attached to the bulky PEGylated nanoliposomes.

Through nanodrop UV spectrophotometer, we observed that 1 µl of free AS1411 contained 758.04 ± 55 ng/µl of ssDNA at 260 nm and compared this with the equivalent amount of AS1411 from the diluted solution of Apt-NLCs (we had added 20 µl of 100 µM AS1411 aptamer to 20 mg of PEG-NLCs to get 5 µM AS1411 in sample) depicted in Fig. 1d. The ssDNA concentration reading in Apt-NLCs was 666.01 ± 03 ng/µl, while non-conjugated plain nanoliposomes (without aptamer conjugation) did not showed any significant absorbance at 260 nm range in nanodrop UV spectrophotometer. This result indicated that more than 80% of AS1411 aptamer used for conjugation was coupled with PEG-NLCs, so 1 mg of PEG-NLCs had capacity to bind approximately 0.20 µM of AS1411 of ss DNA.

The modification ratio of aptamer conjugation to nanoparticle surface may not affect in vivo or in vitro performances of Apt-NLCs. Since single or more aptamers bound to nucleolin receptors would have a similar fate of receptor-mediated cellular internalization. However, the aptamer's binding probability to the nucleolin receptors would obviously be more or faster if the number of aptamers conjugated on the NLC surface is more.

### Physicochemical characterization for test nanoliposomes

**Fig. 2:**
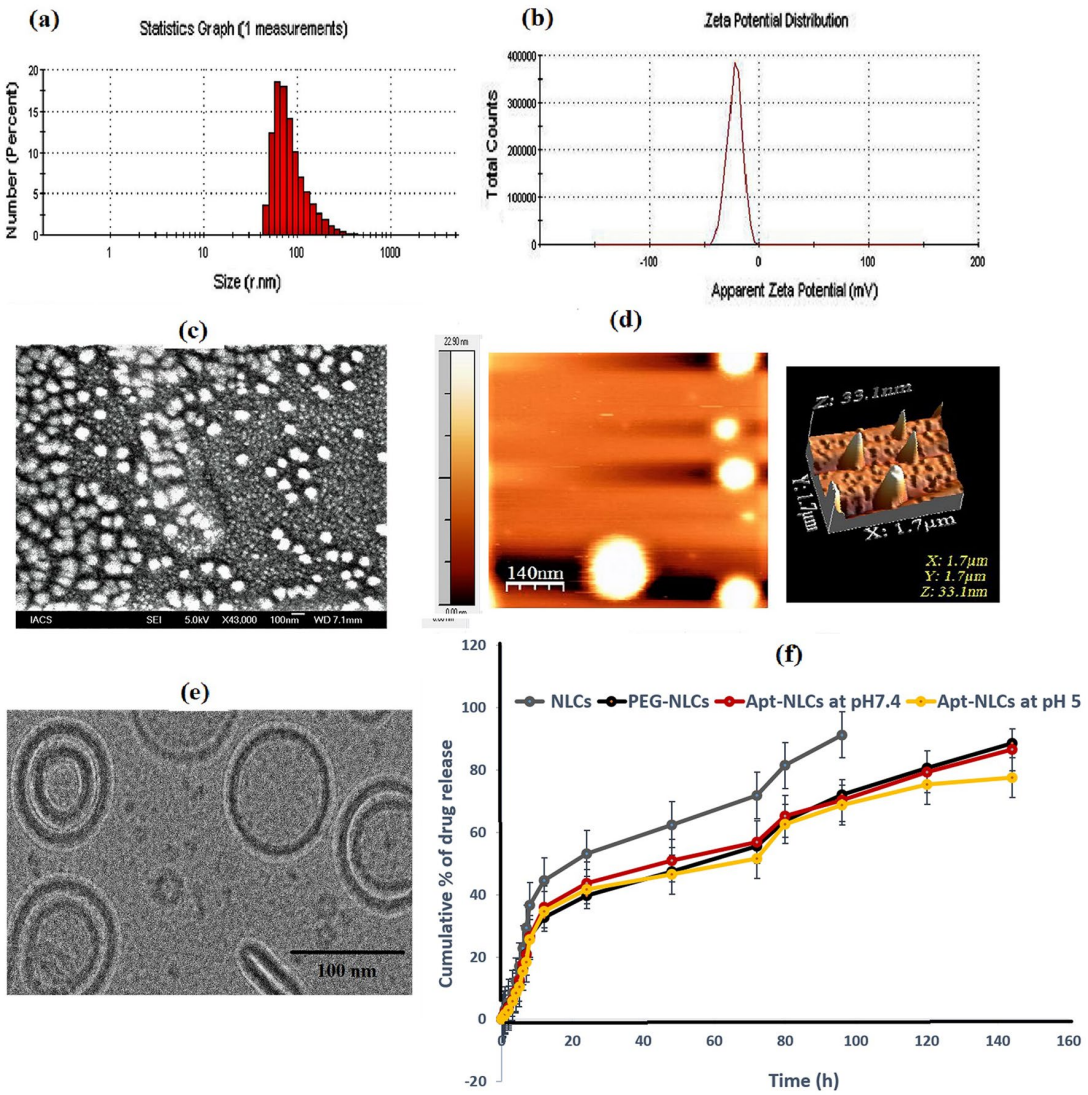
Characterization of aptamer conjugated nanoliposomes Apt-NLCs. **a** average particle size distribution, and **b** Zeta potential, **c** surface morphology applying FESEM images at 43 000 ×. **d** Data by atomic force microscopy. **e** Cryo-TEM image depicts internal morphology, **f** Cumulative % drug release against time for Apt-NLCs as compared NLCs/PEG-NLCs. Data show mean ± standard deviation applying three different experimental values

The mean particle size, PDI, ζ-potential, drug loading, and encapsulation efficacy were optimized for NLCs, PEG-NLCs, and Apt-NLCs. NLC had an average diameter size of 30 nm (Additional file 1: Table S1), which increased to 140 nm (Additional file 1: Table S1) and 150 nm, respectively, upon PEGylation (PEG-NLCs) and PEGylation followed by aptamer conjugation (Apt-NLCs). (Fig. 2). The average surface charge (ζ-potential) was found to be 1.16 mV, − 55.9 mV, and − 22.4 mV for NLCs, PEG-NLCs, Apt-NLCs, respectively (Additional file 1: Table S1 and manuscript Fig. 2). The presence of DSPE PEG-2000-COOH in the liposome has provided a negative charge, which has been decreased for using amino-terminated aptamer conjugation. PDI values (Additional file 1: Table S1) for the respective formulations were 0.251, 0.256, and 0.316, referring to the formulations as uniformly distributed and could possess minimal aggregation in deionized water [22]. Respective drug loading for NLCs, PEG-NLCs, and Apt-NLCs (4.59 ± 0.02%, 4.38 ± 0.04%, and 4.33 ± 0.05%) along with drug entrapment efficiencies in case of all the optimized experimental nanoliposomes (NLCs, PEG-NLCs, and Apt-NLCs) were estimated in Additional file 1: Table 1. Satisfactory drug entrapment efficacy (> 85%) in the experimental nanoliposomes was determined depicted in Additional file 1: Table S1.

**Table 1:** Plasma and liver pharmacokinetic parameters in the HCC rats treated with NLCs/PEG-NLCs/Apt-NLCs and free apigenin as iv bolus form through tail vain

		Api	NLCs	PEG-NLCs	Apt-NLCs
In vivo plasma pharmacokinetic data	Cmax (ng/ml)	15.5 ± 0.16	17.2 ± 1.13	18.1 ± 0.55	19.2 ± 0.51
	AUC last (ng/h/ml)	125.8 ± 52	496.4 ± 62.9	826.4 ± 53^#^	986.2 ± 47^#^
	AUMC	1608 ± 1419	15,290 ± 3089	30,457 ± 2350	37,988 ± 1994
	MRT (h)	12.81 ± 1.58	30.83 ± 1.09	36.94 ± 0.41^$^	38.91 ± 0.21^$^
	AUC 0-∞ (ng h/ml)	130.61 ± 53.4	539.64 ± 79.34	1072.47 ± 171.21*	1446.21 ± 182.65*
	t_1/2_ (h)	5.25 ± 0.25	13.51 ± 0.2	39.02 ± 0.2	43.02 ± 0.25
	Clearance (L/h/Kg)	15.9 ± 0.55	4.03 ± 0.63	2.42 ± 0.66	2.03 ± 0.52
In vivo liver pharmacokinetic data	Cmax (ng/ml)	160 ± 7.87	165.7 ± 7.37	178.9 ± 8.14	223.4 ± 6.64
	Tmax (h)	1 ± 0.15	3 ± 0.2	6 ± 0.22	6 ± 0.26
	AUC last (ng.h/ml)	1828 ± 194	4336 ± 474	9410 ± 553	14,658 ± 592
	AUMC	25,535 ± 3589	119,549 ± 17,352	330,328 ± 2126*	582,753 ± 21,258*
	MRT (h)	13.2 ± 3.8	27.55 ± 6.1	35.48 ± 5.9	42 ± 4.6
	AUC 0-∞ (ng.h/ml)	1898 ± 197	6440 ± 542	8961 ± 705	12,955 ± 892
	T1/2 (h)	5.65 ± 0.52	16 ± 1.2	34 ± 2	50 ± 1.45
	Clearance (L/h/Kg)	1.01 ± 0.01	0.461 ± 0.033	0.213 ± 0.006^	0.136 ± 0.005^

Data from the three independent experiments denote mean ± standard deviation (n = 3)

^#, $^Indicated significant (p < 0.05) improvement in AUC and MRT values in plasma for Apt-NLCs/PEG-NLCs treated animals in comparison to NLCs. (*) Indicated significant (p < 0.05) improvement in AUC in liver values in Apt-NLCs/PEG-NLCs treated animals in comparison to NLCs. ^ indicated significant (p < 0.05) reduction in hepatic clearance in Apt-NLCs/PEG-NLCs treated animals in comparison to NLCs

Indicated physiochemical characterizations were suitable for both in vitro and in vivo drug applications, particularly for hepatocellular drug delivery [43]. Mean zeta potential values (− 22 mV) in the case of Apt-NLCs referred to their stability when dispersed in aqueous suspension for drug administration [44].

### The in-vitro drug release profile of NLCs, PEG-NLCs, and Apt-NLCs with follow-up kinetics

In vitro cumulative percentages of drug release (Fig. 2) for NLCs, PEG-NLCs and Apt-NLCs were plotted against time in PBS (7.4) containing (0.1% W/V) β-cyclodextrin. Here, we observed all the formulations initially upheld almost similar rapid drug release patterns up to 12 h. Later phase PEG-NLCs and Apt-NLCs exhibited a prolonged drug release profile compared to NLCs, most likely because of the PEG coating present outside the test liposomes. NLCs took 92 h to display 90% of cumulative drug release in β-cyclodextrin-PBS medium, whereas PEG-NLCs and Apt-NLCs took 144 h for almost 90% drug release. Further, drug release at acetate buffer media (pH 5) also showed a similar pattern for PEG-NLCs and Apt-NLCs depicted in Additional file 1: Fig. 2.

The drug release data were tested on different kinetic models. The regression coefficient (*R*^2^) values for each Zero-order Kinetics, First-order kinetics, Korsmeyer–Peppas kinetics, Hixson–Crowell, and Higuchi kinetics were elaborated in (Additional file 1: Table S2). The data suggest that drug release from the prepared formulations best fit with the Higuchi kinetic model (*R*^2^ = 0.9811) and specified release component (n) value in derived from Korsmeyer–Peppas kinetic equation suggested the range (0.81 to 0.86), which indicated non-Fickian type of diffusion and drug release patterns were maintained by all the prepared test nanoliposomes in the used media.

An extended drug release profile showed that it took almost 7 days for 90% of drug release in 1% β-cyclodextrin-PBS media (pH 7) from PEG containing nanoliposomes (with or without aptamer conjugation, Apt-NLCs/PEG-NLCs). This pattern was due to the contributary stealthy property of DSPE-PEG-2000 in the test nanoliposomes, which predicted prolonged circulation time. An almost similar pattern of drug release by Apt-NLCs at lower pH (pH 5) also assumed their accumulation toward the target tumor [45].

### Surface and internal morphology of NLCs/PEG-NLCs and Apt-NLCs

FESEM, AFM, and Cryo-TEM evaluated the surface and internal morphology of the prepared nanovesicles. FESEM observation for NLCs /PEG-NLCs (Additional file 1: depicted Fig. S1) and Apt-NLCs (Fig. 2) suggested that the vesicles were nanosized (20–120 nm) with a smooth surface and homogenously well-distributed. Further, the physical architecture of the particles in a three-dimensional mode in AFM revealed that the average mean height for Apt-NLCs was 21.20 ± 0.05 nm (Fig. 2). The thick and intact uni- and bi-lamellar PEG-coated-oligo functionalized surface morphology for Apt-NLCs having a particle size within 100 nm range was clearly visible upon Cryo-TEM observation (Fig. 2). These types of morphological characteristics ensured their stability, cellular accumulation, and sustainability during in vitro and in vivo drug application [19].

### Stability testing

FESEM studies for Apt-NLCs were conducted after 6 months of storage. When refrigerated at − 4 °C, the samples did not portray any major distinguishable changes as compared with freshly prepared Apt-NLCs, while storing at 40 ± 2 °C and 75 ± 5% RH, the morphology differed (Additional file 1: Fig. S2 and Table S3). Further, through estimating drug loading and zeta potentials, it revealed that the Apt-NLCs remained equally potent at refrigerated conditions on 6-month storage (depicted in Additional file 1: Table S3). Still, differences were present when stored at 40 ± 2 °C and 75 ± 5% RH setting. Hence, the formulations were absolutely stable over the period of 6 months in refrigerated conditions.

### In vitro cellular studies

#### In vitro antiproliferative activity

**Fig. 3:**
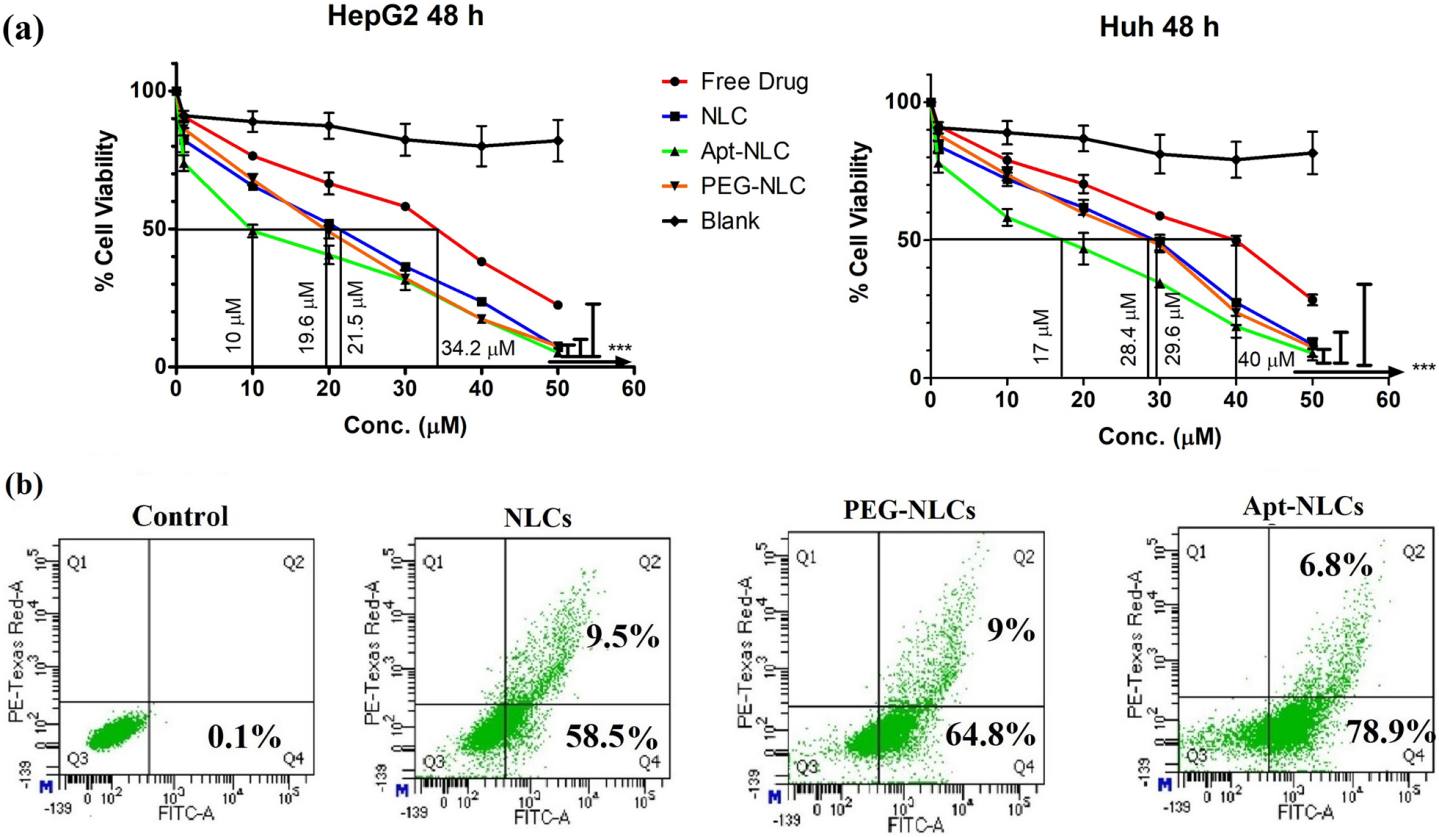
In vitro cytotoxicity and apoptosis analysis. **a** Viability assay in HepG2 and Huh-7 cells after treating them with free drug (apigenin), different experimental formulations (NLCs, PEG-NLCs, and Apt-NLCs), and AS1411 functionalized blank liposomes for 48 h with drug concentration range (10-100 µM) respectively. **b** FACS analysis of cellular apoptosis applying Annexin V-FITC staining in HepG2 cells treated with NLCs, PEG-NLCs, and Apt-NLCs with their equivalent IC50 doses of the drug at 48 h

We investigated in vitro antiproliferative profile of free drug, NLCs, PEG-NLCs, Apt-NLCs, and blank test nanoliposomes against two different types of liver cancer cell lines (Hep G2 and Huh-7 cell lines) over a range of concentrations (1–100 μM) following 48 h incubation period (Fig. 3). We found a similar trend of cytotoxic profile in order of IC_50_ values, as Apt-NLCs < PEG-NLCs < NLCs < free drug < blank in both the cell lines (Fig. 3a). The results reflected the highest cytotoxic potential of aptamer conjugated nanoliposomes (Apt-NLCs) toward liver cancer cells compared to free drug or other nonconjugated nanoliposomes. A negligible cytotoxic activity produced by aptamer-conjugated blank nanoliposomes revealed that neither the aptamer nor the carrier nanoliposome had any interference on the cytotoxicity of Apt-NLCs. The lowest IC_50_ value, 10.1 μM, was observed for Apt-NLCs, against HepG2_,_ while the corresponding IC_50_ value of Apt-NLCs toward Huh-7 was 17.1 μM. So, we have proceeded with Hep G2 cell lines for further in vitro studies to evaluate the apoptotic potential of aptamer-conjugated nanoliposomes over nonconjugated nanoliposomes. Additionally, insignificant cytotoxicity toward normal Human peripheral blood mononuclear cells, PBMC (> 50 μM) proved that Apt-NLCs were suitable to use as a delivery system through the blood to target HCC (Depicted in Additional file 1: Table S4).

#### Apoptosis

We have quantified the apoptotic potential of the experimental nanoliposomes by applying Annexin V-FITC staining using the flow cytometric method. Hep G2 cells were exposed to the nanoliposomes (NLCs, PEG-NLCs, and Apt-NLCs) with their equivalent IC_50_ drug doses for 48 h. The treated group with aptamer functionalized liposomes (Apt-NLCs) showed impressive improvement in the percentage of apoptosis (78.9% in the early and 6.8% in the late phases, a total 85.6%) compared to nonconjugated groups (NLCs/PEG-NLCs) (Fig. 3). NLCs and PEG-NLCs portrayed a total of 68% and 74% of apoptosis, respectively.

This indicated the antitumor potential of apigenin increased even at a low concentration (IC_50_ value in Hep G2) when delivered through an aptamer functionalized nanoformulations.

#### Receptor-mediated cellular uptake

**Fig. 4:**
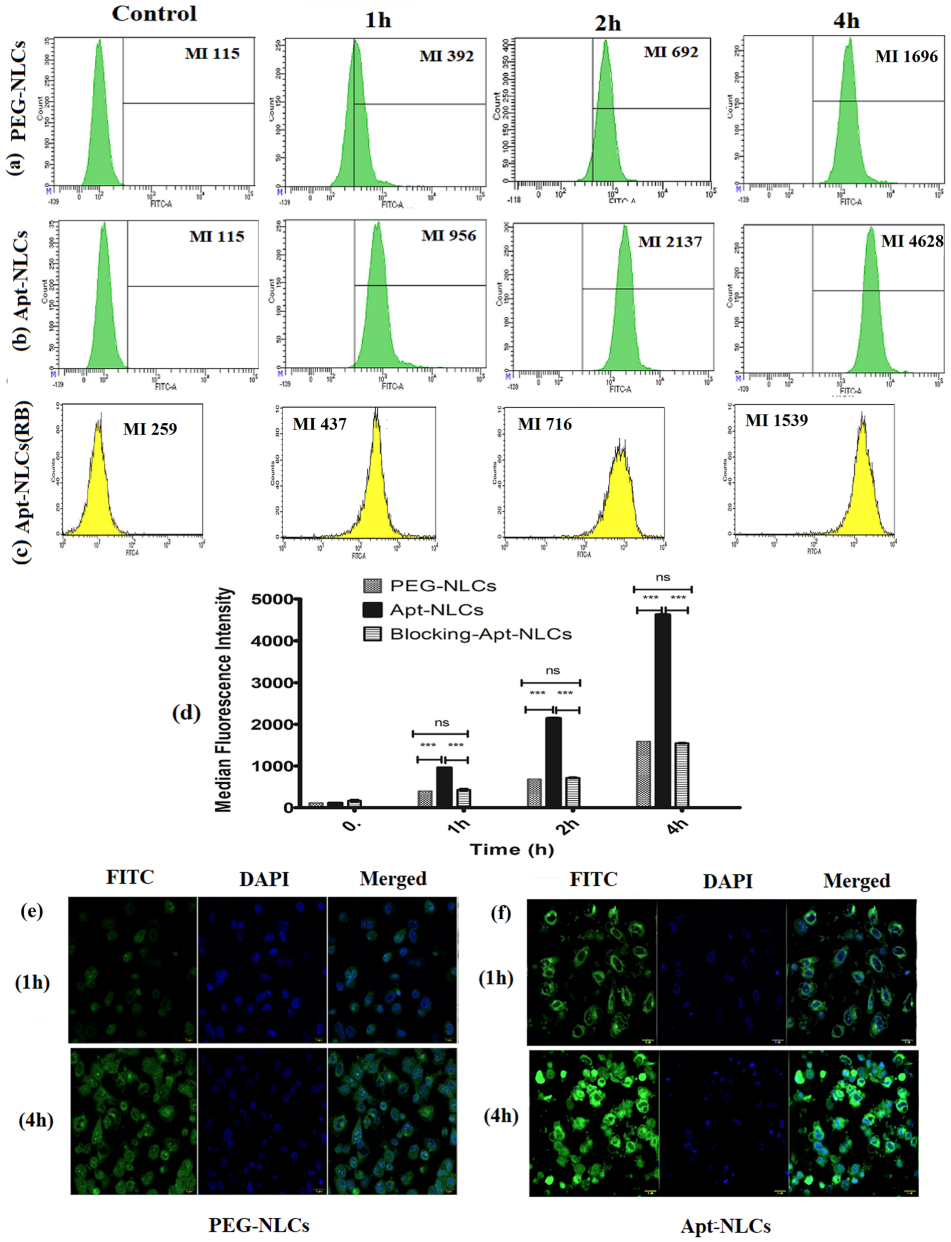
Cellular uptake studies. Flow cytometric data of cellular uptake. **a** PEG-NLCs, **b** Apt-NLCs, **c** Apt-NLCs in the presence of free AS1411 (receptor blocking condition) in Hep G2 cells at 1h, 2h, and 4h **d** Histogram representation of FACS mean fluorescence values obtained through above-mentioned uptake studies. Data represents mean ± SD, (n=3), bar (-) indicates groups between which the comparisons were made. ns: statistically insignificant, *** refer statistical significance at a level of P<0.05. **e** and **f** Confocal microscopic images of cell uptake of PEG-NLCs and Apt-NLCs in Hep G2 at 1h and 4h (Green color shows for FITC-labelled experimental nanoliposomes, PEG-NLCs/ Apt-NLCs, and blue color indicates nucleus stained by DAPI

We estimated uptake of FITC-labelled nanoliposomes, PEG-NLCs and Apt-NLCs (with their equivalent IC_50_ doses of the drug), in Hep G2 cells quantitatively through the flow cytometric method at different time intervals. Parallelly, we run a set with FITC-Apt-NLCs treated HepG2 cells pretreated with an excess amount of free aptamer (AS1411) denoted as a receptor blocking study. There was no considerable difference in mean fluorescence intensity in the case of normal (without the pretreatment) Hep G2 cells treated with PEG-NLCs and in the receptor-blocked Hep G2 cells (using aptamer pretrearment) treated with Apt-NLCs. In contrast, there was a significant improvement in uptake values in the case of normal Hep G2 cells treated with Apt-NLC in a time-dependent manner (Fig. 4a–d). These flow cytometric analyses strongly supported our hypothesis that aptamer (AS1411) functionalized nanoliposomes (Apt-NLCs) augmented cellular uptake in comparison with the nonconjugated nanoliposome (PEG-NLCs), most likely by the nucleolin receptor-facilitated endocytosis as in the cytotoxicity and apoptosis study, we observed notable superior antiproliferative potencial of aptamer-conjugated nanoliposomes (Apt-NLCs) over the nonconjugated nanoformulations. FITC-Apt-NLCs showed much superior cellular uptake in Hep G2 cells at 1 h and 4 h in comparison with PEG-NLCs during confocal microscopy (Fig. 4e and f).

In this competitive cellular uptake study, we tried to understand the probable receptor-mediated endocytosis approach for cell uptake. We observed significant improvement of in vitro uptake of Apt-NLCs (aptamer conjugated PEGylated liposomes) in Hep G2 cells as compared to uptake of Apt-NLCs in free-aptamer pretreated HepG2 cells. Also, bio-ligand AS1411 already reported as a biosensor aptamer had shown a tremendously high affinity toward the carcinogenic biomarker nucleolin receptor of HCC cells [46]. Here, AS1411 aptamer possibly facilitated nucleolin-dependent superior cellular uptake of Apt-NLCs to Hep G2 cells.

#### Cell cycle arrest and comparative estimation of apoptosis-related proteins

**Fig. 5:**
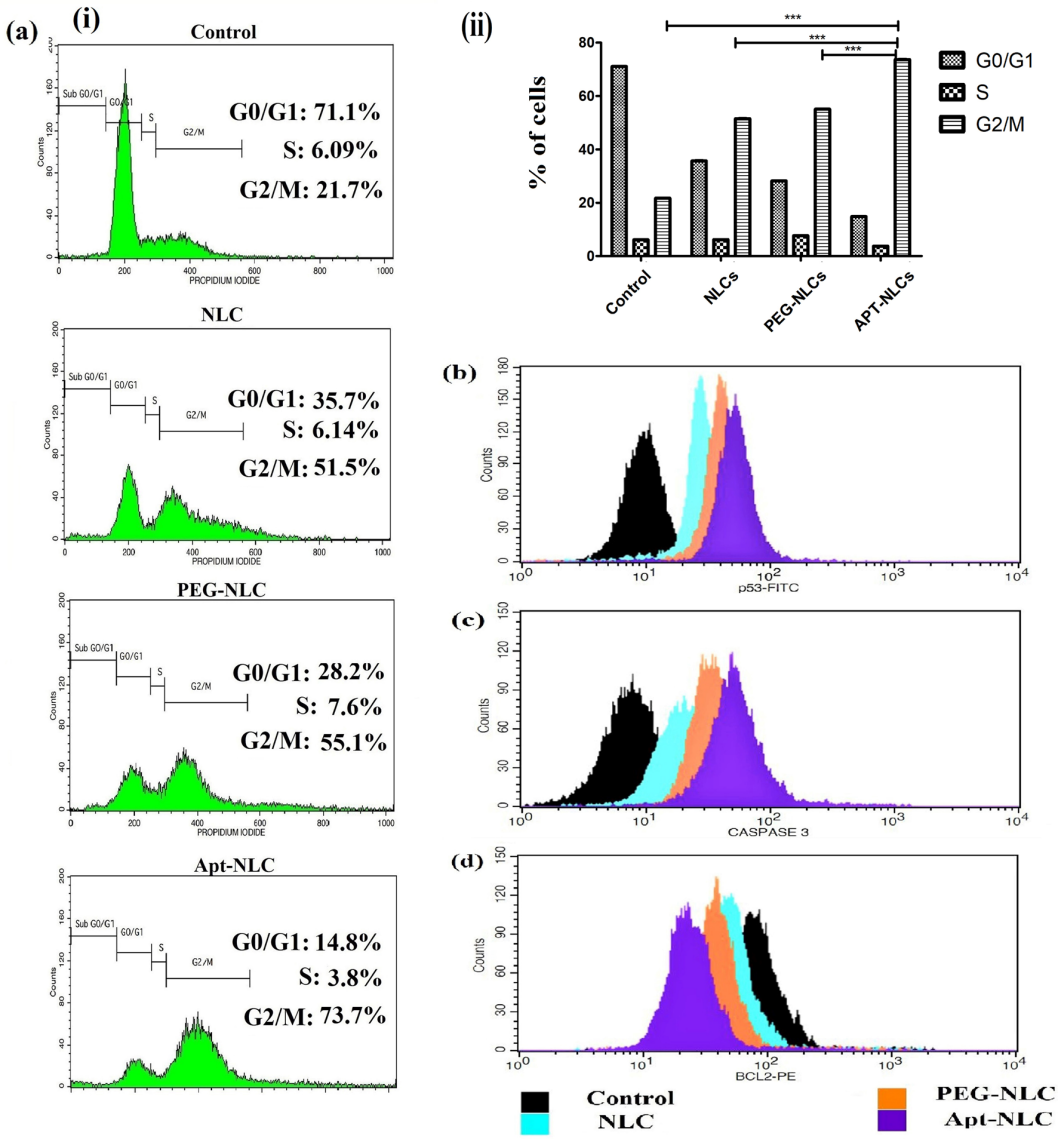
Flowcytometric representation of cell cycle analysis and apoptotic protein expressions studies in Hep G2 cells. **a** i & ii Cell cycle analysis of different experimental nanoliposomes NLCs, PEG-NLCs and Apt-NLCs (highest improvement in G2/M phase in aptamer conjugated nanoliposomes, Apt-NLCs in comparison to non-conjugated nanoliposomes, NLCs/PEG-NLCs. Data represents mean ± SD, (n=3), *** refer statistical significance at a level of P<0.05. **b**, **c** and **d** p53, Casapae-3, and Bcl-2 protein quantifications upon NLCs, PEG-NLCs, and Apt-NLCs treatments, respectively, using FACS

Arresting cell cycle by modulating the phases of cell propagation was postulated as the experimental nanoliposomes (NLCs, PEG-NLCs, Apt-NLCs) caused DNA damage in Hep G2 cells during cellular apoptosis. After flow cytometric analysis, we found that there was a distinguishable increase in the G2/M (73.1%) phase with some changes in the S phase in the cells treated with Apt-NLCs compared to NLCs (51.5%) and PEG-NLCs (55.1%) treatments (Fig. 5). This clearly suggests the enhanced antitumor potential of apigenin while delivered through receptor-targeted drug delivery of nano-system, Apt-NLCs. The damage or repair in DNA during apoptotic processes (either extrinsic or intrinsic) is closely monitored with the different apoptotic-related proteins. Flow cytometric estimation of tumor suppressor proteins, such as p53, caspase, and anti-apoptotic proteins, such as Bcl-2 was conducted. The study depicted the highest mean values for p53 and caspase-3 expressions and the lowest Bcl-2 values with Apt-NLCs compared to other nonconjugated formulations (NLCs/Peg-NLCs) in Hep G2 cells (Fig. 5). Arresting cell cycle at G2/M phase through upregulation in p53 and caspase activities and downregulation of Bcl-2 activity strongly indicates the cellular apoptotic potential of apigenin.

Apigenin allowed apoptosis in neoplastic cells following several apoptotic signaling pathways (extrinsic and Intrinsic), where tumor suppressor protein p53 and anti-apoptotic proteins Bcl-2 (Bcl-XL, Bcl-W, etc.) are very much associated with initiating the apoptotic process [5].

In contrast, caspase activation (majorly caspase-3, caspase-8, and caspase-9) and breakdown or damage in DNA irreversibly play a central role in promoting apoptosis in the tumor cells [47]. Here, the noticeable advanced apoptotic event of arresting p53-mediated cell cycle propagation by apigenin delivered through Apt-NLCs was observed as compared with non-functionalized plain nanoliposomes. This suggests that the modified drug delivery system (Apt-NLCs) boosted the apoptotic potential of apigenin through significant in vitro drug accumulation in the immunomodulatory Hep G2 cells by regulating corresponding signaling pathways irreversibly, thus, strongly checking apoptotic precedence of Apt-NLCs over the nonconjugated nanoliposomes.

### In vivo studies

#### Plasma and liver pharmacokinetic study

**Fig. 6:**
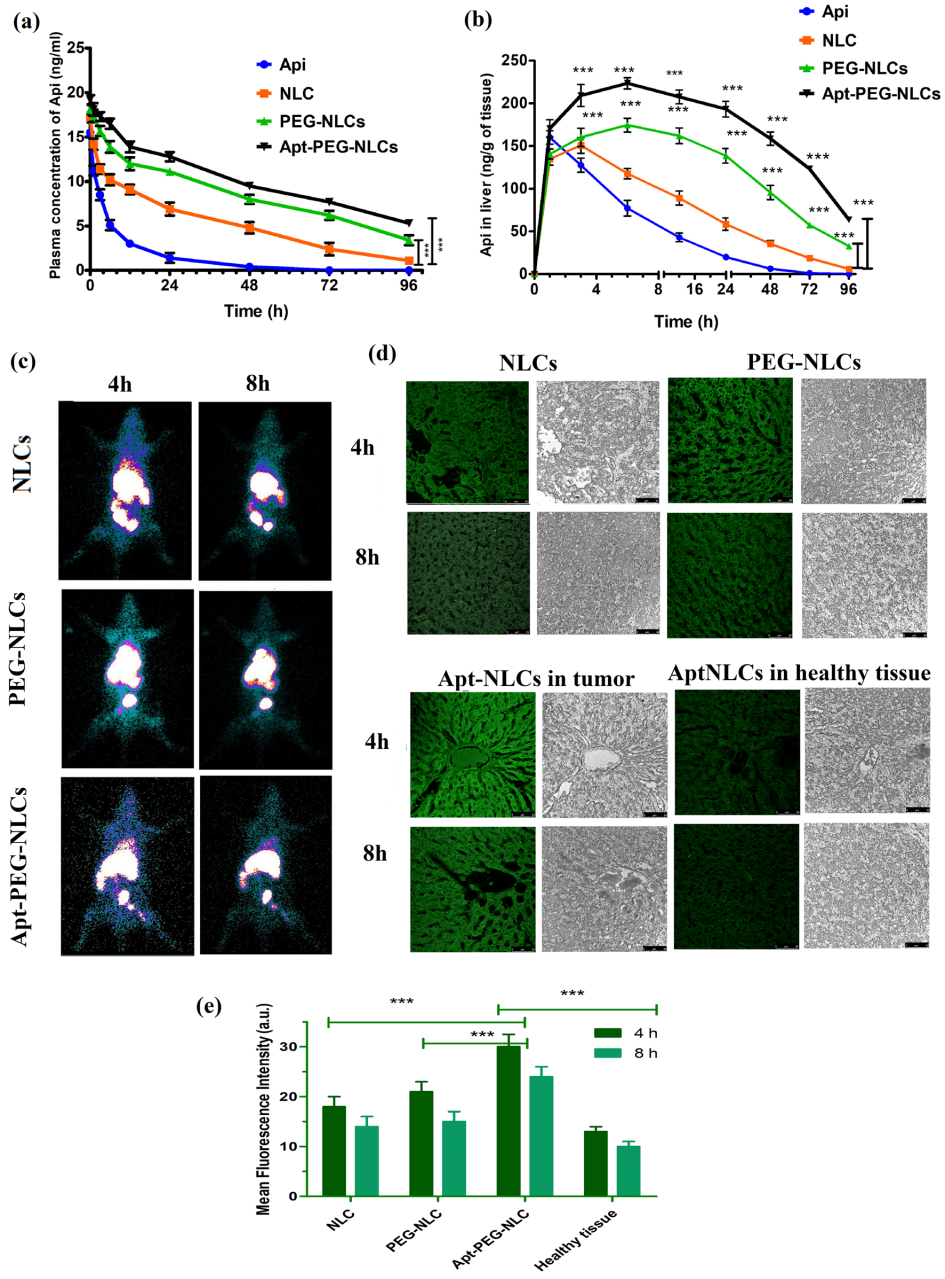
Plasma and liver pharmacokinetics of free drug and all the test formulations (NLCs, PEG-NLCs and Apt-NLCs), biodistribution by gamma-scintigraphy studies, and accumulation of FITC-labelled test nano formulations in cancerous and normal hepatic tissues. **a** Plasma concentration of apigenin vs. time, upon iv administration of NLCs, PEG-NLCs and Apt-NLCs in HCC induced rats, respectively (Data from the three independent experiments show mean ± standard deviation (n = 3); **b** liver concentration of apigenin vs. time curve, upon iv administration of NLCs, PEG-NLCs and Apt-NLCs in HCC induced rats, respectively (Data from the three independent experiments show mean ± standard deviation (n = 3). (***) significant (P< 0.05) improvement in plasma and liver drug concentration in PEG-NLCs and Apt-PEG-NLCs applied animal Gr in comparison to NLCs over the specific time point as indicated in figure; **c** γ Scintigraphy imaginings of HCC rats at 4h and 8h after injecting 99mTc-labeled – (NLCs, PEG-NLCs and Apt-NLCs) through venous cannulation process. **d** Confocal microscopic observation of tumor tissues sections of carcinogenetic rats upon the treatment of FITC-labeled – (NLCs, PEG-NLCs, and Apt-NLCs) at4h and 8 h of administration of iv injection. The figure showed green fluorescence of FITC labeled formulations within the cancerous tissue. The right column showed the photos of the same tissue sections without fluorescence. **e** a comparative study of fluorescence level in both tumor tissue and healthy tissue by FITC-Conjugated Apt-NLCs, also in tumor tissue by FITC-conjugated NLCs/PEG-NLCs

Amounts of apigenin in plasma and liver at predetermined time intervals were estimated after injecting free-apigenin, NLCs, PEG-NLCs, and Apt-NLCs (2 mg apigenin or equivalent dose of formulations /kg body weight) through the tail vein, and the corresponding bio-samples were collected and analyzed using LC–MS/MS method [17]. After plotting the data (representing) graphically, the pharmacokinetic parameters were derived for the experimental nanoliposomes along with the free drug (Fig. 6a). All the nanoformulations maintained a steady drug plasma concentration up to 96 h in contrast with a negligible amount of free-apigenin in plasma at 48 h. However, there were significant improvements in plasma t_1/2_ (~ 3.5-fold), AUC (~ twofold), and MRT (~ 1.2-fold) values in PEG-NLCs and Apt-NLCs treated animals compared to NLCs treated animals were observed. Plasma drug clearance was reduced by up to 50% in the case of both PEG-NLCs and Apt-NLCs compared to NLCs. Further, hepatic apigenin accumulation was highest in the case of Apt-NLCs, and it was increased by 1.2-fold and threefold compared to PEG-NLCs and NLCs, respectively. Elevated hepatic MRT value and reduced hepatic clearance (Table 1) in the case of Apt-NLCs compared to PEG-NLCs and NLCs strongly suggest a greater hepatic accumulation of apigenin when administered as an encapsulated form through a target-specific aptamer mediated-PEG containing drug delivery system.

In vivo, animal models play a vital role in understanding the drug-delivery features and their efficacy in the physiological systems [48]. Here, in vivo pharmacokinetic evaluation of the nanoliposomes (NLCs/PEG-NLCs/Apt-NLCs) in HCC-induced experimental animals (SD, rats) revealed Apt-NLCs carried the highest Plasma half-life (t_1/2_) and AUC. Apt-NLCs also exhibited maximum hepatic drug deposition over more extended periods with the highest MRT values as compared with the nonconjugated nanoliposomes NLCs/PEG-NLCs. They endorsed the lowest drug clearance in HCC-positive experimental rats. All of these superior pharmacokinetic parameters directed prolong and optimum drug bioavailability for apigenin in system as because PEGylated nanoliposomes might improve the circulation time and sustainability of tumor surrounding areas [49].

#### Drug biodistribution study by γ-scintigraphy

After injecting ^99m^Tc-NLCs/^99m^Tc-PEG-NLCs/^99m^Tc-Apt-NLCs in the carcinogen-treated animals, radiolabeled nanoliposomes accumulation was visualized through γ-scintigraphy at 4 h and 8 h of post-injection with estimating bio-distribution of the different experimental nanoliposomes among the different organs of the body. In γ-scintigraphy images, the radio signals were visible in most of the peritoneal region in animals treated with the experimental nanoliposomes, NLCs, and PEG-NLCs, at 4 h, although exclusive and maximum hepatic signals were initiated in Apt-NLCs treated animals (60.69 ± 1.63% ID/g in liver tissue) at 8 h (Table 2, Fig. 6b). Further, at 8 h, a time-dependent drug distribution pattern was observed. Clear and predominant hepatic restricted signals were noticed in Apt-NLCs treated animals (49.51% ID/g of tissue) (Table 2), whereas, NLCs/PEG-NLCs offered indistinct drug accumulation (18.89 %ID/g and 33.078% ID/g of tissue, respectively) in the liver, along with considerable detectable signals in stomach, lung, kidney and to the other adjacent tissues of the body (Table 2). This could mention selective hepatic accumulations of aptamer functionalized nanoliposomes over nonconjugated formulations in vivo, most likely after following the receptor-mediated uptake of Apt-NLCs through the attachment of aptamer, AS1411 to the target oncogenic biosensor nucleolin receptor overexpressed on neoplastic hepatocytes.

**Table 2:** Results of biodistribution of experimental formulations

Organ/Tissues	Biodistribution of ^99m^Tc-labeled experimental nanoliposomes in HCC induced rat model					
	^99m^Tc-NLCs		^99m^Tc-PEG-NLCs		^99m^Tc-Apt-NLCs	
	4 h	8 h	4 h	8 h	4 h	8 h
Heart	0.587 ± 0.043	0.347 ± 0.065	0.489 ± 0.047	0.309 ± 0.028	0.199 ± 0.026	0.155 ± 0.007
Blood	2.538 ± 0.067	1.484 ± 0.091	5.8973 ± 0.53	2.369 ± 0.366	4.392 ± 0.201	2.523 ± 0.115
Liver	37.234 ± 0.33	18.899 ± 1.25	47.686 ± 1.92^**#**^	33.078 ± 1.06	60.69 ± 1.63*	49.51 ± 0.478^
Lung	3.778 ± 0.209	2.554 ± 0.411	2.502 ± 0.295	2.678 ± 0.135	1.667 ± 0.349	2.319 ± 0.260
Spleen	0.627 ± 0.033	0.545 ± 0.036	0.833 ± 0.047	0.633 ± 0.106	0.724 ± 0.052	0.446 ± 0.042
Muscle	0.163 ± 0.013	0.142 ± 0.013	0.1893 ± 0.01	0.172 ± 0.022	0.118 ± 0.024	0.156 ± 0.011
Intestine	13.902 ± 0.29	8.988 ± 0.932	17.974 ± 0.61	14.27 ± 0.249	5.828 ± 0.074	3.651 ± 0.048
Stomach	0.487 ± 0.018	0.342 ± 0.028	0.653 ± 0.039	0.355 ± 0.012	0.387 ± 0.016	0.308 ± 0.032
Kidney	10.033 ± 0.71	4.461 ± 0.465	4.475 ± 0.276	3.254 ± 0.127	3.631 ± 0.406	2.591 ± 0.268
Urine	15.616 ± 0.14	14.706 ± 0.24	10.702 ± 0.82	9.755 ± 0.769	6.929 ± 0.851	5.654 ± 0.650

Data are expressed in % mean of injected dose (ID) per gram of organ/tissue ± SD (n = 3)

(#) Indicated significant (p < 0.05) amount of drug accumulation in liver in PEG-NLCs treated Gr over NLCs, while (*) indicated significant (p < 0.05) amount of drug accumulation in liver in Apt-PEG-NLCs treated Gr over PEG-NLCs Gr. at 4 h. (^) indicated significant (p < 0.05) amount of drug accumulation in liver in Apt-PEG-NLCs treated Gr over PEG-NLCs at 8 h

All these data indicated the rationally designed aptamer (AS1411) functionalized PEGylated nanoliposomes offered significant drug retention (Apt-NLCs > PEG-NLCs/NLCs) selectively in the carcinogenic liver due to the uptake of aptamer (AS1411) conjugated nanoliposomes (Apt-NLCs) by the neoplastic hepatocytes. Most likely, it followed aptamer (AS1411) sensitive biomarker nucleolin receptor-mediated cellular uptake. Thus, the synergic effect of precise drug accumulation and their sustainability in neoplastic hepatic tissues reinforced the dominance of Apt-NLCs as the finest as well as promising drug delivery vehicles in vivo.

### Intratumor deposition of FITC-labeled nanoliposomes

We investigated the neoplastic hepatic area after injecting FITC-labeled nanoliposomes into HCC-positive animals to compare the uptake of different test nanoliposomes within the neoplastic tissue and their surrounding liver tissues. We visualized a distinctive higher fluorescence in the neoplastic hepatic tissue of the Apt-NLCs injected animals at different time points compared to the surrounding liver tissues (Fig. 6e). At the same time, much less localized fluorescence was observed in tumors treated with the other nanoformulations than Apt-NLCs in HCC animals. It suggests the superiority and specificity of AS1411 conjugated drug delivery toward the HCC tumors.

### Macroscopic and microscopic examinations of the liver of rats treated with experimental nanoliposomes

**Fig. 7:**
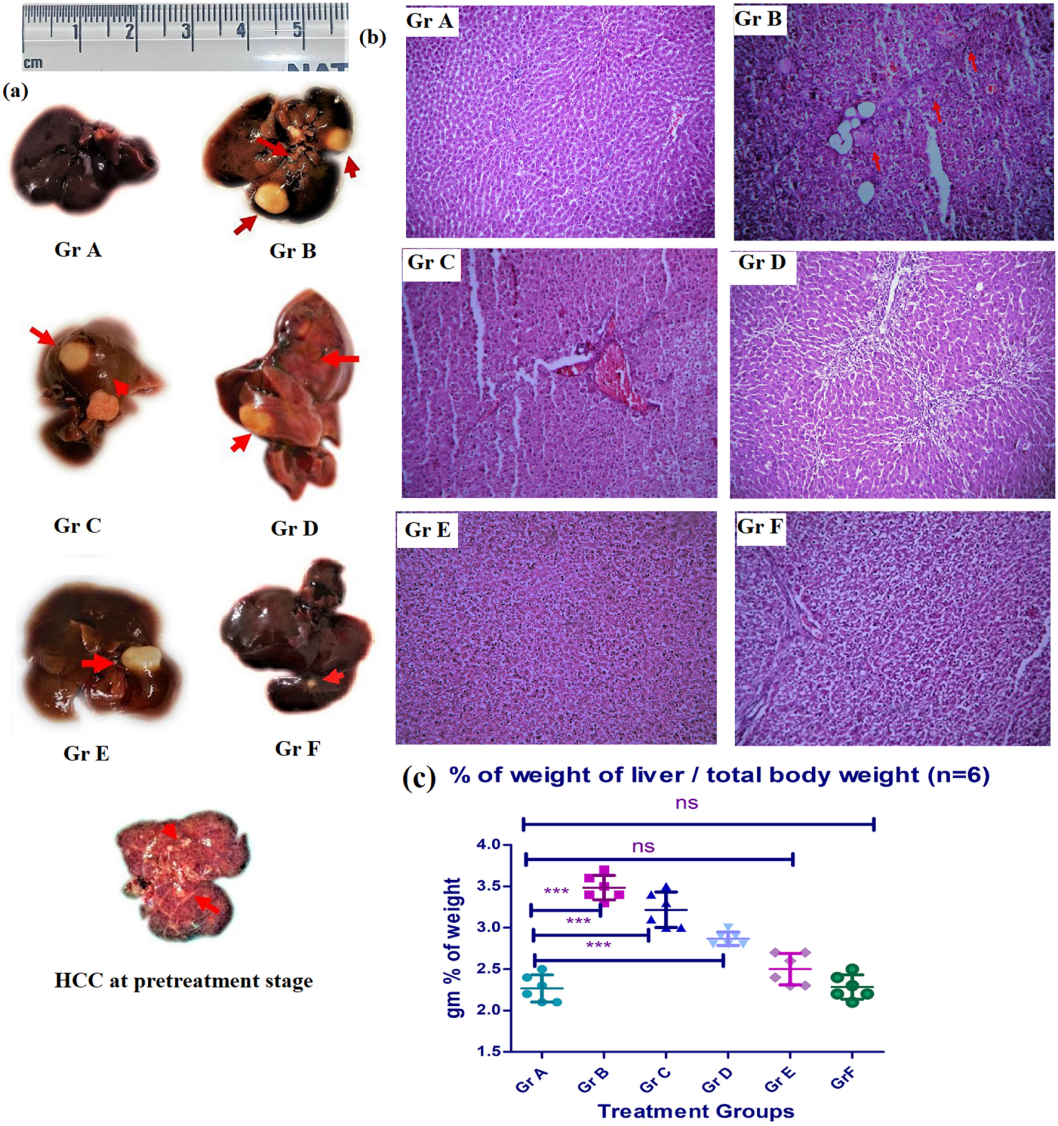
Microscopic and macroscopic hepatic analysis of the experimental rats. **a** Macroscopic (in 100X magnification) liver images for the different experimental animal groups, Gr A to Gr F. **b** H & E histopathological microscopic images of liver tissue sections for various animal groups. **c** Represented % of weight of liver vs total body weight in different treatment group. (***) represent significant (p < 0.05) changes among group as indicated in figure and ns denoted as non-significant changes

One or multiple tumorigenic hyperplastic nodules (HN) with different tumor volumes were developed in experimental animals receiving carcinogens and various drug formulations. Post-treatment, we observed Gr B animals (HCC control) showed enormous tumorigenic growth (Fig. 7). There was no gross improvement of tumor suppression noticed upon free apigenin treatment in Gr C animals. In the case of nonconjugated nanoliposome (NLCs/PEG-NLCs) treated groups (Gr D & E), an almost 50–60% reduction in average tumor volume was detected in comparison to the control group. Liver weight against total body weight of animals varied upon the experimental treatments (Fig. 7c). However, the value was close to normal in the case of carcinogen-treated rats treated with Apt-NLCs. Nevertheless, a significant diminishing effect in tumor incidences (> 90%) in the case of Gr F animals was noticed compared to other treated groups. The carcinogen-treated animals (Gr F) that received Apt-NLCs comprised some tiny tumor lesions with an average size of < 50 mm^3^, confirming the superior therapeutic potential of Apt-NLCs as a commanding anticancer formulation.

We have done an extensive microscopic examination of hepatic tissues collected from all the treatment group animals at the end of the treatment protocol. In histopathological images (Fig. 7), we observed characteristic HAF lesions with lobular or spongy inflammatory ducts in the case of Gr B, carcinogen control animals. However, upon anticancer treatment with Api/NLCs/PEG-NLCs/Apt-NLCs, various degrees of improvement in reforming typical hepatocellular architecture were observed. The appearance of scattered apoptotic artifacts in the case of group D & E animals was observed. In the case of Group F animals, Apt-NLCs promoted restructuring the cellular panache almost toward normal hepatocytes. The highest degree of reduction in hepatic altered focal lesions (HAF) occurrence (Fig. 7) was observed in the case of Apt-NLCs (Gr F), along with a significant reduction of tumor volume as well as HAF area (Table 3) compared to NLCs/PEG-NLCs (Gr D & Gr E) treated experimental animals, which also supported the optimum curative potential of aptamer-conjugated nanoliposomes rather than other normally developed nonconjugated nanoformulations (NLCs/PEG-NLCs).

**Table 3:** Quantitative data referring tumor development and total HAF (hepatic altered foci) area among different group of experimental animals

Treatment Groups	Number of the rat developed tumour post treatment	Average tumour volume (n = 6) (mm)^3^	Number of HAF area per cm^2^ under microscopic observation on treatment
Gr A	0/6	–	–
Gr B	6/6	1502.89 ± 9.98^#^	86.74 ± 5.90^#^
Gr C	6/6	1261.5 8 ± 8.09^#$^	67.89 ± 8.09^#^^
Gr D	5/6	821.08 ± 4.67^#$^	56.89 ± 6.06^#^^^ns^
Gr E	3/6	786.73 ± 5.56^#$ns^	49.97 ± 0.58^#^^**
Gr F	2/6	35.89 ± 7.56^#$^***	12.63 ± 0.43^#^^***
Gr G	0/6	–	–

^#^Data represented mean ± SD (where, n = 6 in each group of animals)

^$^^,^^ indicated significant (p < 0.05) reduction of tumor volume and HAF observed in (Gr C-Gr F) in comparison to positive control group Gr- B. Again, compared among the test nanoliposome (Gr D, Gr E, and Gr F), in Gr F (aptamer conjugated nanoliposome treated group) significant (p < 0.05) reduction of both tumor volume and HAF were observed mentioned in figure (***); while in Gr E (PEG-NLCs treated group showed non-significant (ns) amount of tumor volume reduction, (**) significant amount of HAF reduction and Gr D (NLCs treated animal group) showed non-significant (ns) amount of HAF reduction

### Assaying apoptosis-related signaling proteins in the experimental rats

**Fig. 8:**
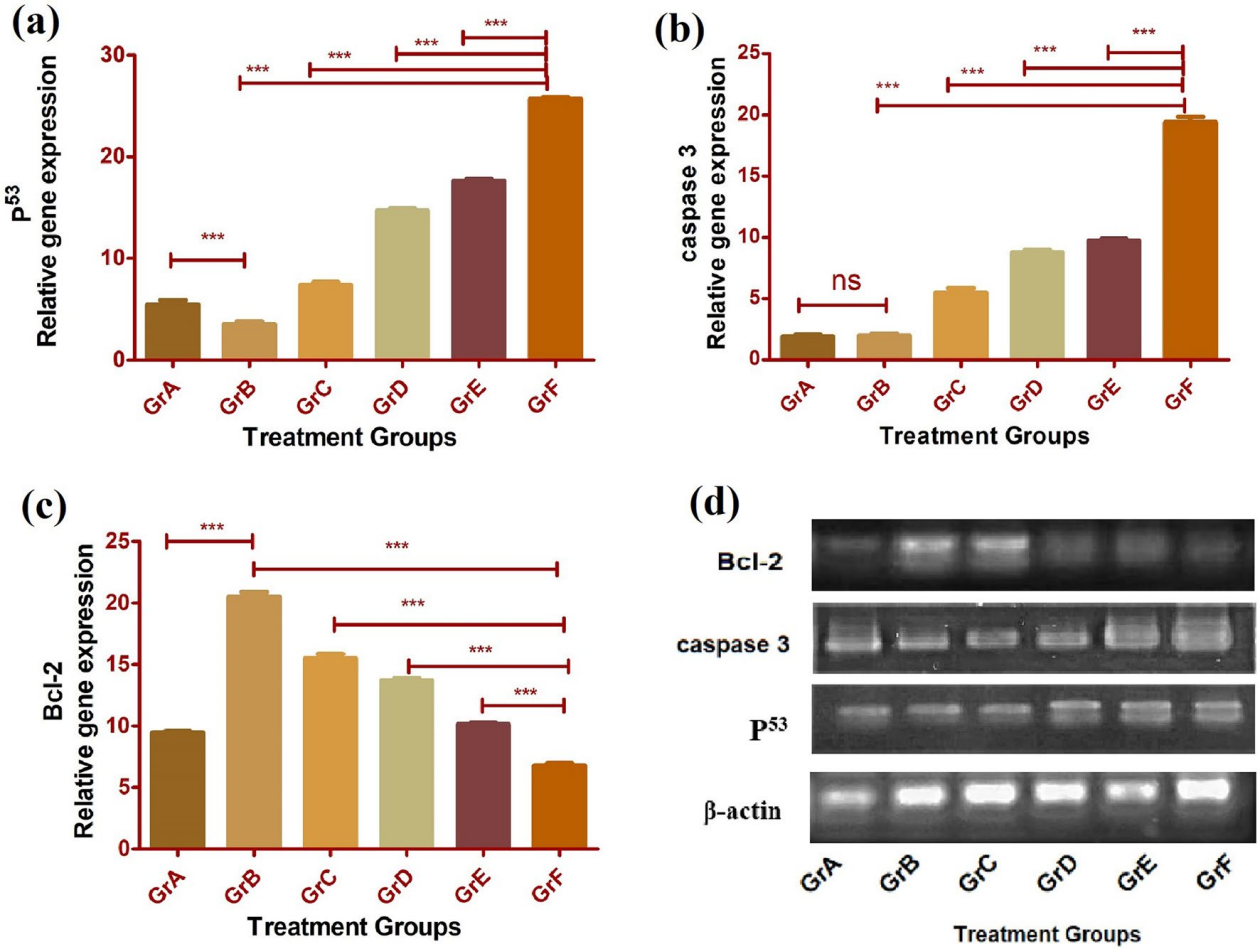
Comparative apoptotic gene expression analysis through RT-PCR with liver tissue samples from different experiential groups of animals (A-F). **a** Representing increases level of p53, **b** caspase-3, and **c** decreased level of Bcl-2 expression. Data indicated ± SD (n=3), (***) expressed significant (p < 0.05) upregulation or downregulation in gene, in conjugated nanoliposomes Apt-NLCs treated animals (GR F), samples in comparison to non-conjugated NLCs/ PEG-NLCs nanoliposomes (Gr D/F), free apigenin (Gr C) and carcinogen positive animals (Gr B). ns represented no significant changes among Gr A and Gr B for caspase expression assay. **d** Representing β- actin, p53, caspase -3, Bcl-2 gene expression of different experiential groups of animals in agarose gel electrophoresis study

Histograms (Fig. 8) representation from the data generated through RT-PCR assay with liver tissue samples obtained from different experimental groups of rats expressed the maximal upregulation of p53, caspase-3 activation, and the maximum downregulation in Bcl-2 in carcinogen-induced rats treated with Apt-NLCs (functionalized nanoliposomes), suggesting the highest degree of apoptosis caused by Apt-NLCs in comparison to nonconjugated nanoliposomes (NLCs/PEG-NLCs).

### Assaying hepatic functionality in different groups of experimental animals

Serum AST, ALT, and ALP levels generally indicate normal or pathological conditions of the liver [27]. Here, serum AST, ALT, and ALT were increased in carcinogen-treated (carcinogen control) rats compared to the normal rats. Otherwise, the levels were decreased upon treatment of NLCs/PEG-NLCs and Apt-NLCs. (Additional file 1: Table S5). The data showed the highest level of improvement in Gr F animals (Apt-NLCs treated carcinogenic animals). Moreover, no significant changes in normal serum AST, ALT, and ALP values in Gr G animals (normal animals treated with Apt-NLCs).

Higher bioavailability and intratumor drug delivery by aptamer conjugated nanoliposomes, Apt-NLCs, in experimental animals produced the greater anticancer potential of apigenin than other plain nanoliposomes (NLCs/Apt-NLCs). Likewise, predominant and significant therapeutic progress was observed in HCC-developed rats treated with Apt-NLCs by controlling tumor incidences in the liver and restoring HAF toward normal in H&E-stained liver tissue sections over the nonconjugated nanoformulations and free apigenin. Further, in vivo apoptosis-related protein expression estimation among the different groups of experimental rats using qRT-PCR revealed the highest activities in caspase-3, caspase-9, p53, and the lowest activity in Bcl-2 in Apt-NLCs treated animals (Fig. 8). There were not many changes observed in liver functionality (ALT, AST, ALP) in Apt-NLCs treated in normal animals. The findings, thus, support the remarkable opportunity of aptamer functionalized PEG-containing nanoliposomes (Apt-NLCs) as a potent drug delivery system that could deliver chemotherapeutics, importantly active bio-flavonoids (here apigenin) to the target neoplastic hepatic region.

## Conclusion

Achievement of any chemotherapeutic or any bio-active compounds in cancer therapy depends on their effective and rational drug delivery approach. Bioflavonoids, as a lipophilic anticancer agent, have to overcome robust pharmacokinetic as well as molecular drug delivery challenges in HCC. In our present study, we have developed aptamer functionalized nanoliposomes and highlighted first time the mechanistic approach of drug uptake, accumulation, and modulation in apoptotic signaling pathways for apigenin in neoplastic hepatic cells, which was triggered by reasonably designed aptamer functionalized PEG-containing nanoliposomes. Phosphorothioated amino-modified AS1411 aptamer-conjugated apigenin-loaded PEG-NLCs successfully induced apoptosis in Hep G2 liver cancer cells and arrested cell-cycle mostly at G2/M phase by upregulation in p53 and caspase activities and downregulation of Bcl-2 activity. Accumulating the aptamer conjugated formulation (Apt-NLCs) was distinctively more in the liver than in the other tissues. Further, Apt-NLCs accumulation in tumors in the liver was predominantly greater than in the surrounding non-cancerous hepatic tissue, suggesting successful site-specific drug distribution. Apt-NLC reduced tumor incidences and neoplastic hepatic altered lesions, suggesting its potential anticancer effect in vivo. Apt-NLCs impressively targeted the neoplastic hepatic region in rats. Further, we can explore the functional efficacy of aptamer functionalized nanoliposomes in additional preclinical animal models for gathering more relevant scientific pieces of evidence. Thus, the process for translation from preclinical to clinical drug development will be narrow.

## Supplementary Information

**Additional file 1.**Table S1: Details of physicochemical characterization of NLCs, PEG-NLCs, Apt-NLCs: Table for particle size, drug loading and zeta potential of plain nanoliposomes (NLCs), PEGylated Nanoliposomes (PEG-NLCs) and Aptamer functionalized PEGylated nanoliposomes (Apt-NLCs); 2. Fig S1: Particle size and surface characterization of plain nanoliposomes (NLCs) and PEGylated Nanoliposomes (PEG-NLCs). (a), (c) average particle size distribution for NLCs and PEG-NLCs respectively, (b), (d) surface morphology applying FESEM images for NLCs and PEG-NLCs respectively, 3. Table S2: In vitro drug release kinetics: The kinetic equations of drug release data tested for NLCs/PEG-NLCs/Apt-NLCs on various kinetic models with corresponding R2 (Regression coefficient) values were studied; 4. Fig. S2: Stability studies: (a) FESEM image of Apt-NLCs on (-4 °C) storage, (b) FESEM image of Apt-NLCs on (40 ± 2°C and 75 ± 5% RH), Table-S3: Drug loading and zeta potential of Apt-NLCs stored at (-4 °C) and (40 ± 2°C and 75 ± 5% RH) for six months; 5. Table S4: Cytotoxicity studies by MTT-assay: Table depicted respective IC50 (µM), Half maximum inhibitory concentration for apigenin, NLCs, PEG-NLCs, Apt-NLSs, Apt-BNLCs in HepG2 cells, Huh-7 cells and PBMC cells, 6. Table-S5: Assaying hepatic functionality in different groups of experimental animals # Data represented mean ± SD (where, n=6 in each group of animals). Table depicted respective AST/ALT/ALP values in all the experimental carcinogenetic animal groups treated with apigenin (Gr C), plain nanoliposomes (Gr D), PEGylated nanoliposomes (Gr E), aptamer conjugated PEGylated nanoliposomes (Gr F) along with normal animals treated with normal saline (Gr A) and normal animal treated with aptamer conjugated nanoliposomes.

## References

[1] Llovet JM, Kelley RK, Villanueva A, Singal AG, Pikarsky E, Roayaie S, Lencioni R, Koike K, Zucman-Rossi J, Finn RS (2021). Hepatocellular carcinoma. Nat Rev Dis Prim.

[2] Oiseth SJ, Aziz MS (2017). Cancer immunotherapy: a brief review of the history, possibilities, and challenges ahead. J Cancer Metastasis Treat..

[3] Yan S, Zhao P, Yu T, Gu N (2019). Current applications and future prospects of nanotechnology in cancer immunotherapy. Cancer Biol Med.

[4] Dou XQ, Wang H, Zhang J, Wang F, Xu GL, Xu CC, Xu HH, Xiang SS, Fu J, Song HF (2018). Aptamer-drug conjugate: targeted delivery of doxorubicin in a HER3 aptamer-functionalized liposomal delivery system reduces cardiotoxicity. Int J Nanomedicine.

[5] Belfiore L, Saunders DN, Ranson M, Thurecht KJ, Storm G, Vine KL (2018). Towards clinical translation of ligand-functionalized liposomes in targeted cancer therapy: challenges and opportunities. J Control Release.

[6] Yan X, Qi M, Li P, Zhan Y, Shao H (2017). Apigenin in cancer therapy: anti-cancer effects and mechanisms of action. Cell Biosci.

[7] Bhattacharya S, Mondal L, Mukherjee B, Dutta L, Ehsan I, Debnath MC, Gaonkar RH, Pal MM, Majumdar S (2018). Apigenin loaded nanoparticle delayed development of hepatocellular carcinoma in rats. Nanomedicine.

[8] Mahmoudi S, Ghorbani M, Sabzichi M, Ramezani F, Hamishehkar H, Samadi N (2019). Targeted hyaluronic acid-based lipid nanoparticle for apigenin delivery to induce Nrf2-dependent apoptosis in lung cancer cells. J Drug Deliv Sci Technol..

[9] Moosavian SA, Sahebkar A (2019). Aptamer-functionalized liposomes for targeted cancer therapy. Cancer Lett.

[10] Yazdian-Robati R, Bayat P, Oroojalian F, Zargari M, Ramezani M, Taghdisi SM, Abnous K (2020). Therapeutic applications of AS1411 aptamer, an update review. Int J Biol Macromol.

[11] Volk DE, Lokesh GLR (2017). Development of phosphorothioate DNA and DNA thioaptamers. Biomedicines.

[12] Hussain Z, Khan S, Imran M, Sohail M, Shah SWA, de Matas M (2019). PEGylation: a promising strategy to overcome challenges to cancer-targeted nanomedicines: a review of challenges to clinical transition and promising resolution. Drug Deliv Transl Res.

[13] Aden DP, Fogel A, Plotkin S, Damjanov I, Knowles BB (1979). Controlled synthesis of HBsAg in a differentiated human liver carcinoma-derived cell line. Nature.

[14] Moscato S, Ronca F, Campani D, Danti S (2015). Poly(vinyl alcohol)/gelatin hydrogels cultured with HepG2 cells as a 3D model of hepatocellular carcinoma: a morphological study. J Funct Biomater.

[15] Mersch-Sundermann V, Knasmüller S, Wu XJ, Darroudi F, Kassie F (2004). Use of a human-derived liver cell line for the detection of cytoprotective, antigenotoxic and cogenotoxic agents. Toxicology.

[16] Şenyildiz M, Kilinc A, Ozden S (2018). Investigation of the genotoxic and cytotoxic effects of widely used neonicotinoid insecticides in HepG2 and SH-SY5Y cells. Toxicol Ind Health.

[17] Faghihzadeh F, Anaya NM, Schifman LA, Oyanedel-Craver V (2016). Fourier transform infrared spectroscopy to assess molecular-level changes in microorganisms exposed to nanoparticles. Nanotechnol Environ Eng.

[18] Dey NS, Mukherjee B, Maji R, Satapathy BS (2016). Development of linker-conjugated nanosize lipid vesicles: a strategy for cell selective treatment in breast cancer. Curr Cancer Drug Targets.

[19] Mashreghi M, Zamani P, Moosavian SA, Jaafari MR (2020). Anti-epcam aptamer (Syl3c)-functionalized liposome for targeted delivery of doxorubicin: in vitro and in vivo antitumor studies in mice bearing C26 colon carcinoma. Nanoscale Res Lett.

[20] Alibolandi M, Ramezani M, Abnous K, Hadizadeh F (2016). AS1411 aptamer-decorated biodegradable polyethylene glycol-poly (lactic-co-glycolic acid) nanopolymersomes for the targeted delivery of gemcitabine to non-small cell lung cancer in vitro. J Pharm Sci.

[21] Moosavian SA, Abnous K, Badiee A, Jaafari MR (2016). Improvement in the drug delivery and anti-tumor efficacy of PEGylated liposomal doxorubicin by targeting RNA Aptamers in mice bearing breast tumor model. Colloids Surf B Biointerfaces.

[22] Dutta D, Chakraborty A, Mukherjee B, Gupta S (2018). Aptamer-conjugated apigenin nanoparticles to target colorectal carcinoma: a promising safe alternative of colorectal cancer chemotherapy. ACS Appl bio-Mater.

[23] Li L, Hou J, Liu X, Guo Y, Wu Y, Zhang L, Yang Z (2014). Nucleolin-targeting liposomes guided by aptamer AS1411 for the delivery of SiRNA for the treatment of malignant melanomas. Biomaterials.

[24] Wang MS, Reed SM (2012). Direct visualization of electrophoretic mobility shift assays using nanoparticle-aptamer conjugates. Electrophoresis.

[25] Yuce M, Kurtb H (2017). How to make nanobiosensors: surface modification and characterisation of nanomaterials for biosensing applications. RSC Adv.

[26] Peng L, Liang Y, Zhong X, Liang Z, Tian Y, Li S, Liang J, Wang R, Zhong Y, Shi Y, Zhang X (2020). Aptamer-conjugated gold nanoparticles targeting epidermal growth factor receptor variant iii for the treatment of glioblastoma. Int J Nanomed.

[27] Rudra A, Deepa RM, Ghosh MK, Ghosh S, Mukherjee B (2010). Doxorubicin-loaded phosphatidylethanolamine-conjugated nanoliposomes: in vitro characterization and their accumulation in liver, kidneys, and lungs in rats. Int J Nanomed.

[28] Ruozi B, Belletti D, Tombesi A, Tosi G, Bondioli L, Forni F, Vandelli MA (2011). AFM, ESEM, TEM, and CLSM in liposomal characterization: a comparative study. Int J Nanomed.

[29] Paul B, Gaonkar RH, Mukhopadhyay R, Ganguly S, Debnath MC, Mukherjee B (2019). Garcinol-loaded novel cationic nanoliposomes: in vitro and in vivo study against B16F10 melanoma tumor model. Nanomedicine.

[30] Peretz Damari S, Shamrakov D, Varenik M, Koren E, Nativ-Roth E, Barenholz Y, Regev O (2018). Practical aspects in size and morphology characterization of drug-loaded nano-liposomes. Int J Pharm.

[31] Sengupta S, Paul P, Mukherjee B, Gaonkar RH, Debnath MC, Chakraborty R, Khatun N, Roy S (2018). Peripheral nerve targeting by procaine-conjugated ribavirin-loaded dual drug nanovesicle. Nanomedicine.

[32] Dutta L, Mukherjee B, Chakraborty T, Das MK, Mondal L, Bhattacharya S, Gaonkar RH, Debnath MC (2018). Lipid-based nanocarrier efficiently delivers highly water-soluble drug across the blood-brain barrier into brain. Drug Deliv.

[33] Ganguly S, Dewanjee S, Sen R, Chattopadhyay D, Ganguly S, Gaonkar R, Debnath MC (2021). Apigenin-loaded galactose tailored PLGA nanoparticles: a possible strategy for liver targeting to treat hepatocellular carcinoma. Colloids Surf B Biointerfaces.

[34] Farahbakhsh Z, Zamani MR, Rafienia M, Gülseren O, Mirzaei M (2020). In silico activity of AS1411 aptamer against nucleolin of cancer cells. Iran J Blood Cancer.

[35] Liao Z-X, Chuang E-Y, Lin C-C, Ho Y-C, Lin K-J, Cheng P-Y, Chen K-J, Wei H-J, Sung H-W (2015). An AS1411 aptamer-conjugated liposomal system containing a bubble-generating agent for tumor-specific chemotherapy that overcomes multidrug resistance. J Control Release Soc.

[36] Jin X, Yang Q, Zhang Y (2017). Synergistic apoptotic effects of apigenin TPGS liposomes and tyroservatide: implications for effective treatment of lung cancer. Int J Nanomedicine.

[37] Sarkar A, Mukherjee B, Chatterjee M (1994). Inhibitory effect of β-carotene on chronic 2-acetylaminofluorene induced hepatocarcinogenesis in rat: reflection in hepatic drug metabolism. Carcinogenesis.

[38] Chakraborty S, Dlie ZY, Chakraborty S, Roy S, Mukherjee B, Besra SE (2020). Aptamer-functionalized drug nanocarrier improves hepatocellular carcinoma toward normal by targeting neoplastic hepatocytes. Mol Ther Nucleic Acids.

[39] Ghosh MK, Patra F, Ghosh S, Hossain CM, Mukherjee B (2014). Antisense oligonucleotides directed against insulin-like growth factor-II messenger ribonucleic acids delay the progress of rat hepatocarcinogenesis. J Carcinog.

[40] Mabrouk Zayed MM, Sahyon HA, Hanafy NAN, El-Kemary MA (2022). The effect of encapsulated apigenin nanoparticles on HePG-2 cells through regulation of P53. Pharmaceutics.

[41] He X, Sun J, Huang X (2018). Expression of caspase-3, Bax and Bcl-2 in hippocampus of rats with diabetes and subarachnoid hemorrhage. Exp Ther Med.

[42] Wang B, Zhao X-H (2017). Apigenin induces both intrinsic and extrinsic pathways of apoptosis in human colon carcinoma HCT-116 cells. Oncol Rep.

[43] Hoshyar N, Gray S, Han H, Bao G (2016). The effect of nanoparticle size on in vivo pharmacokinetics and cellular interaction. Nanomedicine.

[44] Soema PC, Geert-Jan Willems G, Jiskoot W, Amorij J, Kersten GF (2015). Predicting the influence of liposomal lipid composition on liposome size, zeta potential and liposome-induced dendritic cell maturation using a design of experiments approach. Eur J Pharm Biopharm.

[45] Kanamala M, Palmer BD, Jamieson SM, Wilson WR, Wu Z (2019). Dual PH-sensitive liposomes with low PH-triggered sheddable PEG for enhanced tumor-targeted drug delivery. Nanomedicine.

[46] Reyes-Reyes EM, Teng Y, Bates PJ (2010). A new paradigm for aptamer therapeutic AS1411 action: uptake by macropinocytosis and its stimulation by a nucleolin-dependent mechanism. Cancer Res.

[47] Shendge AK, Chaudhuri D, Basu T, Mandal N (2021). A natural flavonoid, apigenin isolated from clerodendrum viscosum leaves, induces G2/M phase cell cycle arrest and apoptosis in MCF-7 cells through the regulation of P53 and caspase-cascade pathway. Clin Transl Oncol.

[48] Park W, Chen J, Cho S, Park S-J, Larson AC, Na K, Kim D-H (2016). Acidic PH-triggered drug-eluting nanocomposites for magnetic resonance imaging-monitored intra-arterial drug delivery to hepatocellular carcinoma. ACS Appl Mater Interfaces.

[49] Batra J, Robinson J, Mehner C, Hockla A, Miller E, Radisky DC, Radisky ES (2012). PEGylation extends circulation half-life while preserving in vitro and in vivo activity of tissue inhibitor of metalloproteinases-1 (TIMP-1). PLoS ONE.

