# Supplementary material for: Phosphorothioated amino-AS1411 aptamer functionalized stealth nanoliposome accelerates bio-therapeutic threshold of apigenin in neoplastic rat liver: a mechanistic approach
Source: J Nanobiotechnology. 2023 Jan 25;21:28. doi: 10.1186/s12951-022-01764-4 (PMC9875447; doi:10.1186/s12951-022-01764-4)
Supplement: Supplementary file 1 — Additional file 1.Table S1: Details of physicochemical characterization of NLCs, PEG-NLCs, Apt-NLCs: Table for particle size, drug loading and zeta potential of plain nanoliposomes (NLCs), PEGylated Nanoliposomes (PEG-NLCs) and Aptamer functionalized PEGylated nanoliposomes (Apt-NLCs); 2. Fig S1: Particle size and surface characterization of plain nanoliposomes (NLCs) and PEGylated Nanoliposomes (PEG-NLCs). (a), (c) average particle size distribution for NLCs and PEG-NLCs respectively, (b), (d) surface morphology applying FESEM images for NLCs and PEG-NLCs respectively, 3. Table S2: In vitro drug release kinetics: The kinetic equations of drug release data tested for NLCs/PEG-NLCs/Apt-NLCs on various kinetic models with corresponding R2 (Regression coefficient) values were studied; 4. Fig. S2: Stability studies: (a) FESEM image of Apt-NLCs on (-4 °C) storage, (b) FESEM image of Apt-NLCs on (40 ± 2°C and 75 ± 5% RH), Table-S3: Drug loading and zeta potential of Apt-NLCs stored at (-4 °C) and (40 ± 2°C and 75 ± 5% RH) for six months; 5. Table S4: Cytotoxicity studies by MTT-assay: Table depicted respective IC50 (µM), Half maximum inhibitory concentration for apigenin, NLCs, PEG-NLCs, Apt-NLSs, Apt-BNLCs in HepG2 cells, Huh-7 cells and PBMC cells, 6. Table-S5: Assaying hepatic functionality in different groups of experimental animals # Data represented mean ± SD (where, n=6 in each group of animals). Table depicted respective AST/ALT/ALP values in all the experimental carcinogenetic animal groups treated with apigenin (Gr C), plain nanoliposomes (Gr D), PEGylated nanoliposomes (Gr E), aptamer conjugated PEGylated nanoliposomes (Gr F) along with normal animals treated with normal saline (Gr A) and normal animal treated with aptamer conjugated nanoliposomes. [file 12951_2022_1764_MOESM1_ESM.docx]

Supplementary data

**Phosphorothioated amino-AS1411 aptamer functionalized stealth nanoliposome accelerates bio-therapeutic threshold of apigenin in neoplastic rat liver: a mechanistic approach**

**Moumita Dhara^a^, Ashique Al Hoque^a, b^, Ramkrishna Sen^a^, Debasmita Dutta^c, d^, Biswajit Mukherjee^a^*, Brahamacharry Paul^a^, Soumik Laha^e^**.

a Department of Pharmaceutical Technology, Jadavpur University, Kolkata 700032, India.

b Department of Coatings and Polymeric Materials, North Dakota State University, Fargo, United States.

c Dana Farber Cancer Institute, Boston, MA, USA

d Harvard Medical School, Boston, MA, USA

e Central Instrument Facility, CSIR- Indian Institute of Chemical Biology,

Kolkata 700032, India.

*Corresponding Author

Professor (Dr.) Biswajit Mukherjee

Department of Pharmaceutical Technology,

Jadavpur University, Kolkata-700032, India

Telephone: +91-33-2457 2588

Email: biswajit.mukherjee@jadavpuruniversity.in/ biswajit55@yahoo.com

**1. Table S1:** Details of physicochemical characterization of NLCs, PEG-NLCs, Apt-NLCs: Table for particle size, drug loading and zeta potential of plain nanoliposomes (NLCs), PEGylated Nanoliposomes (PEG-NLCs) and Aptamer functionalized PEGylated nanoliposomes (Apt-NLCs)

| Formulation  Type | Composition | % Of  Drug  Loading^a^ | % Of  Loading  Efficiency^a^ | Z-Average  (nm) | Zeta potential(mV) | PDI values |
| --- | --- | --- | --- | --- | --- | --- |
| **NLCs** | D: CHL: SPC  (5:25:70) | 4.59 ± 0.02 | 91.86% | 20 | 1.16 | 0.251±1 |
| **PEG-NLCs** | D: CHL: SPC: DSPE –PEG-2000  (5:25:60:10) | 4.38 ± 0.04 | 87.6% | 100 | -55.9 | 0.262±1.5 |
| **Apt-NLCs** | D: CHL: SPC: DSPE –PEG-2000  (5:25:60:10)  (Functionalized with aptamer, AS1411) | 4.33 ± 0.05 | 86..6% | 155 | -22.4 | 0.316±1.1 |

**Note: aEach value represents mean ± SD (n=3).**

**Abbreviations: D: Drug (apigenin), CHL: Cholesterol, SPC: Soya lecithin.**

S1

**2. Fig S1:**Particle size and surface characterization of plain nanoliposomes (NLCs) and PEGylated Nanoliposomes (PEG-NLCs). (a), (c) average particle size distribution for NLCs and PEG-NLCs respectively, (b), (d) surface morphology applying FESEM images for NLCs and PEG-NLCs respectively.


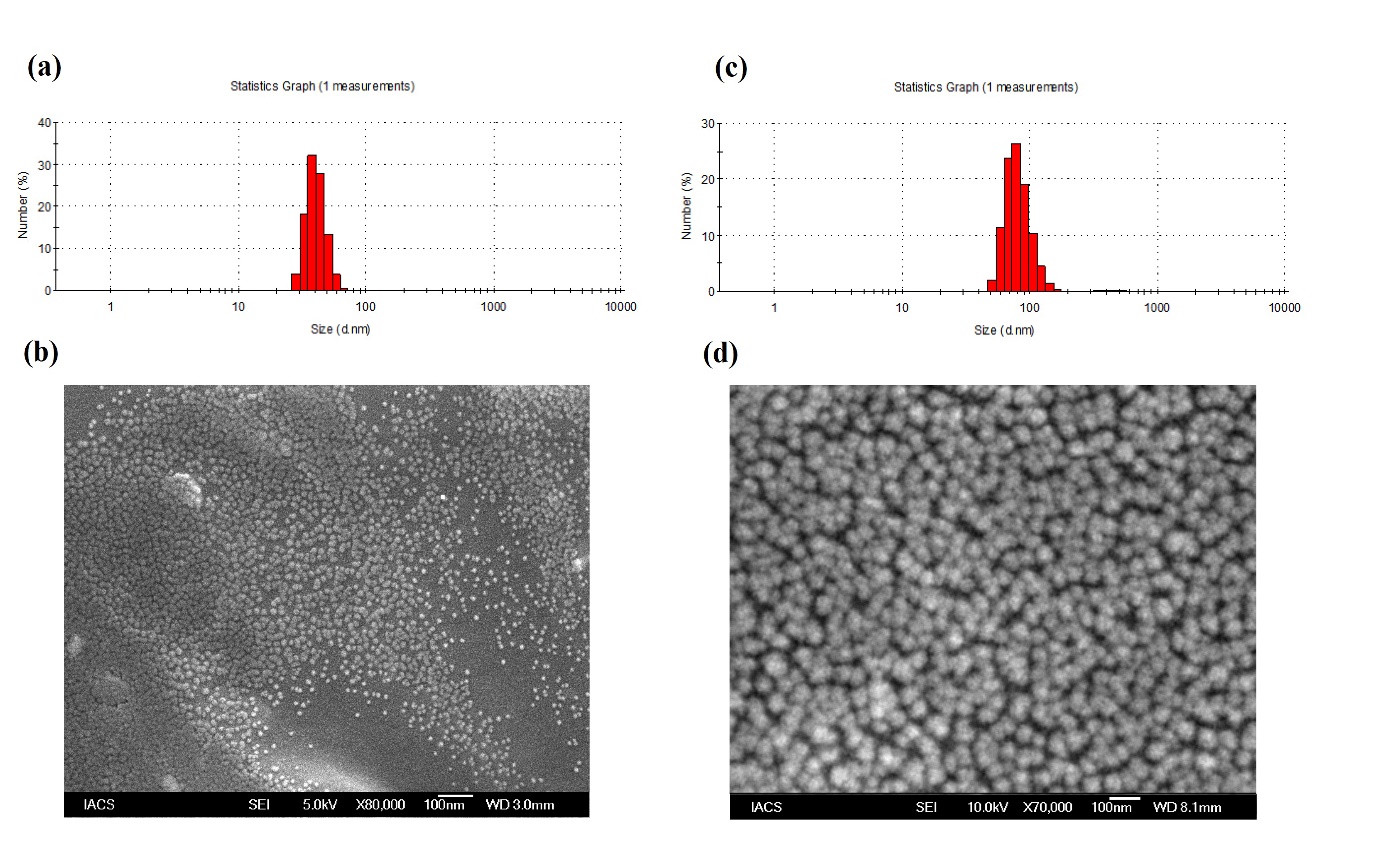


S2

**3. Table S2:** *In vitro* drug release kinetics**:** The kinetic equations of drug release data tested for NLCs/PEG-NLCs/Apt-NLCs on various kinetic models with corresponding *R^2^* (Regression coefficient) values were studied.

| Kinetics models  (in vitro release) | NLCs  *R^2^* Value Kinetic eq^n^ | | PEG-NLCs  *R^2^* Value Kinetic eq^n^ | | Apt-NLCs  *R^2^* Value Kinetic eq^n^ | |
| --- | --- | --- | --- | --- | --- | --- |
| Zero-order | 0.8165 | y = 0.8932x +12.476 | 0.9191 | y = 0.6166x  + 9.996 | 0.8917 | y = 0.6014x  + 11.024 |
| First-order | 0.8615 | y = 0.0092x  + 12.476 | 0.9691 | y = 0.0059x  + 1.9764 | 0.9715 | y = 0.0055x  + 1.9649 |
| Higuchi | 0.958 | y = 0.1006x  + 0.3276 | 0.9811 | y = 0.1297x  + 0.407 | 0.9739 | y = 0.1305x  + 0.3145 |
| Korsmeyer−Peppas | 0.9062 | y + 0.8665x  + 0.4616  (*n* value 0.866) | 0.9287 | y = 0.8169x  + 0.3617  (*n* value 0.817) | 0.9211 | y = 0.8185x  + 0.3695  (*n* value 0.818) |
| Hixson−Crowell | 0.9391 | y = 0.0237x  + 0.1705 | 0.9713 | y = 0.0157x  + 0.1239 | 0.9581 | y = 0.0149x  + 0.0152 |

Note: Each value represents mean ± SD (n=3), *n* value indicates Fickian/non-Fickian diffusion.

S3

**4. Fig. S2:** Stability studies: (a) FESEM image of APT-NLCs on (-4 °C) storage, (b) FESEM image of Apt-NLCs on (40 ± 2°C and 75 ± 5% RH).

On six months of storage, (when refrigerated at 4° C) FESEM studies did not portray abrupt distinguishable changes as compared with freshly prepared Apt-NLCs, while storing at 40 ± 2 °C and 75 ± 5% RH showed morphological disruption in test formulations depicted in Supplementary Figure 2. Further, assay for drug content estimation (drug loading) and zeta potential at respective condition revealed that the Apt-NLCs remained equally potent at refrigerated conditions on six-month storage (depicted in appendices Figure 2). But major differences were present when stored at 40 ± 2 °C and 75 ± 5% RH setting.


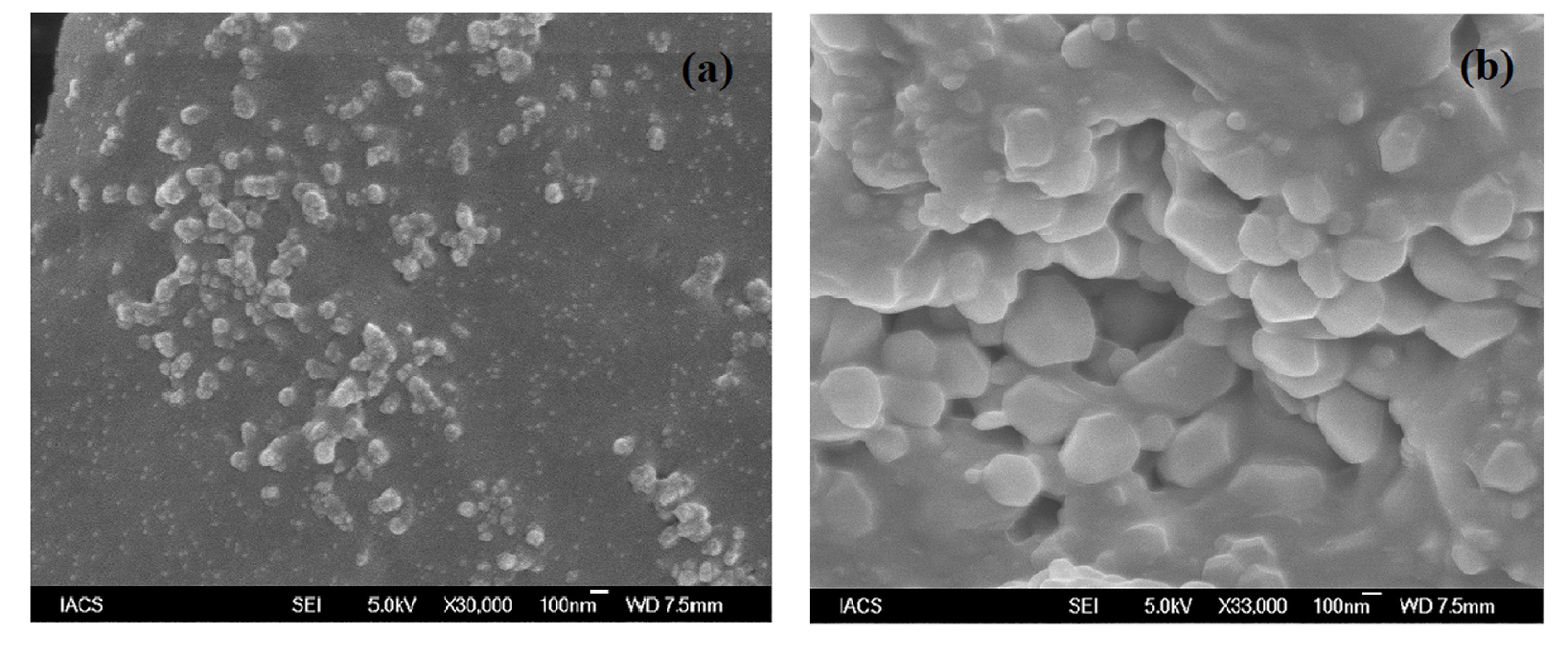


**Table S3:**Drug loading and zeta potential of Apt-NLCs stored at (-4 °C) and (40 ± 2°C and 75 ± 5% RH) for six months.

| **Formulation**  **Type** | **% Of**  **Drug**  **Loading^a^** | **Zeta potential(mV)** |
| --- | --- | --- |
| **Apt-NLCs**  **(-4 °C)** | **4.165 ± 0.04** | **-53** |
| **Apt-NLCs**  **(40 ± 2°C and 75 ± 5% RH)** | **0.771 ± 0.02** | **-1.6** |

**Note: Each value represents mean ± SD (n=3)**

S4

**5. Table S4:** Cytotoxicity studies by MTT-assay: Table depicted respective IC50 (µM), Half maximum inhibitory concentration for apigenin, NLC, PEG-NLCs, Apt-NLSs, Apt-BNLCs in different cell types

| IC50 (µM), Half maximum inhibitory concentration  Sample HepG2 Huh7 PBMC | | | |
| --- | --- | --- | --- |
| Apigenin | **34. ± 0.16** | **40.0 ± 1.74** | **>50** |
| NLC | **21.5 ± 0.60** | **29.6 ± 1.37** | **>50** |
| PEG-NLC | **19.6 ± 0.85** | **28.4 ± 4.54** | **>50** |
| Apt-NLC | **10.0 ± 0.85** | **17.0 ± 3.98** | **>50** |
| Apt-BNLC | **>50** | **>50** | **>50** |

**Note: Each value represents mean ± SD (n=3)**

S5

**6. Table S5:** Assaying hepatic functionality in different groups of experimental animals:

| Treatment groups | Hepatic enzyme parameters  AST (IU/L) ALT (IU/L) ALP (KA units) | | |
| --- | --- | --- | --- |
| Group A | **75± 1.67#** | **26± 1.43#** | **60± 2.19#** |
| Group B | **182± 1.39#** | **68± 2.24#** | **154± 1.33#** |
| Group C | **167± 2.27#** | **53± 1.36#** | **139± 1.59#** |
| Group D | **132± 1.51#** | **41± 2.29#** | **117± 1.73#** |
| Group E | **129± 1.71#** | **34± 1.62#** | **98± 1.87#** |
| Group F | **72± 2.19#** | **25± 1.87#** | **59± 2.35#** |
| Group G | **69± 1.32#** | **22± 1.53#** | **54± 1.74#** |

# Data represented mean ± SD (where, n=6 in each group of animals). Table depicted respective AST/ALT/ALP values in all the experimental carcinogenetic animal groups treated with apigenin (Gr C), plain nanoliposomes (Gr D), PEGylated nanoliposomes (Gr E), aptamer conjugated PEGylated nanoliposomes (Gr F) along with normal animals treated with normal saline (Gr A) and normal animal treated with aptamer conjugated nanoliposomes.

S6
